# Root exudation of contrasting drought-stressed pearl millet genotypes conveys varying biological nitrification inhibition (BNI) activity

**DOI:** 10.1007/s00374-021-01578-w

**Published:** 2021-07-09

**Authors:** Arindam Ghatak, Florian Schindler, Gert Bachmann, Doris Engelmeier, Prasad Bajaj, Martin Brenner, Lena Fragner, Rajeev K. Varshney, Guntur Venkata Subbarao, Palak Chaturvedi, Wolfram Weckwerth

**Affiliations:** 1 Molecular Systems Biology (MOSYS), Department of Functional and Evolutionary Ecology, Faculty of Life Sciences, University of Vienna, Althanstrasse 14, A-1090 Vienna, Austria; 2 Center of Excellence in Genomics and Systems Biology (CEGSB), International Crops Research Institute for the Semi-Arid Tropics (ICRISAT), Patancheru, Hyderabad, Telangana 502324 India; 3 Vienna Metabolomics Center (VIME), University of Vienna, Althanstrasse 14, A-1090 Vienna, Austria; 4 State Agricultural Biotechnology Centre Centre for Crop and Food Innovation, Murdoch University, Murdoch, WA 6150 Australia; 5 Crop, Livestock, and Environment Division, International Research Center for Agricultural Sciences (JIRCAS), Tsukuba, Ibaraki 305-8686 Japan

**Keywords:** Root exudates, Pearl millet, Primary metabolites, Secondary metabolites, LC–MS, GC–MS

## Abstract

Roots secrete a vast array of low molecular weight compounds into the soil broadly referred to as root exudates. It is a key mechanism by which plants and soil microbes interact in the rhizosphere. The effect of drought stress on the exudation process and composition is rarely studied, especially in cereal crops. This study focuses on comparative metabolic profiling of the exudates from sensitive and tolerant genotypes of pearl millet after a period of drought stress. We employed a combined platform of gas and liquid chromatography coupled to mass spectrometry to cover both primary and secondary metabolites. The results obtained demonstrate that both genotype and drought stress have a significant impact on the concentration and composition of root exudates. The complexity and function of these differential root exudates are discussed. To reveal the potential effect of root exudates on the soil microbial community after a period of drought stress, we also tested for biological nitrification inhibition (BNI) activity. The analysis revealed a genotype-dependent enhancement of BNI activity after a defined period of drought stress. In parallel, we observed a genotype-specific relation of elongated root growth and root exudation under drought stress. These data suggest that the drought stress-dependent change in root exudation can manipulate the microbial soil communities to adapt and survive under harsh conditions.

**Supplementary Information:**

The online version contains supplementary material available at 10.1007/s00374-021-01578-w.

## Introduction

Plant root exudates represent a complex mixture of hundreds of compounds, classified into different categories based on their molecular weight and solubility in water (Badri and Vivanco 2009; Baetz and Martinoia 2014; Canarini et al. 2019; Nardi et al. 2020). The quantity and composition of root exudates are species-specific and can be altered through abiotic factors to enhance the ecosystem (Williams and de Vries 2020). Root exudates play an important role in the amelioration of plant stresses (Doornbos et al. 2012), in the increase of soil microbial activity (Mommer et al. 2016), in the biodegradation of pollutants (Jha et al. 2015) and in the uptake of nutrients (Dakora and Phillips 2002; Cesco et al. 2010). It is documented that root exudates account for up to 40% of the photosynthetically fixed C (Doornbos et al. 2012). Root exudation provides a direct chemical communication tool for plants and rhizosphere microbes (Yuan et al. 2015) as well as plant–plant interaction (Mommer et al. 2016). It can influence plant tolerance and survival under severe abiotic stress (Gargallo-Garriga et al. 2018).

Exudates also mediate biological nitrification inhibition (BNI) in the soil. It is a key adaptation strategy of the plant root systems to limit soil-nitrifier activity to reduce nitrate generation and associated nitrogen (N) losses in N-limited soils (Nardi et al. 2020; Subbarao et al. 2006a, b; Subbarao and Searchinger 2021). Biological nitrification inhibition (BNI) has been identified in a wide range of various plant species (Subbarao et al. 2007a); specific BNI (AT units activity g^−1^ root dry weight) ranged from 0 (i.e. no detectable activity) to 18.3 AT units. Among the tested cereal and legume crops, sorghum, pearl millet and groundnut showed detectable BNI in root exudate. The exudate-derived compounds directly involved in BNI are less described. Few successful examples include compounds such as 3-(4 hydroxyphenyl) propionate (MHPP), sakuranetin and sorgoleone, all of which have been identified as nitrification inhibitors in root exudates of sorghum (Subbarao et al. 2013a, 2013b), as well as 1,9-decanediol, an inhibitor that has been found in root exudates of rice (Sun et al. 2016). It has been demonstrated that sorgoleone potentially reduces the abundance of soil nitrifiers (AOB and AOA *amo*A genes) (Coskun et al. 2017a; Subbarao et al. 2013a; Sarr et al. 2020).

Exudates can be sampled in hydroponics or in soil (Oburger and Jones 2018). The collection is easier from hydroponics but the collected exudates have less ecological relevance than those collected from the soil. A soil-based sampling approach is a ‘quick and dirty’ technique where unaltered root exudate targeting the entire root system can be obtained (Oburger and Jones 2018). There are few studies that have investigated soil-based sampling for root exudates after a defined period of drought stress (Gargallo-Garriga et al. 2018). Recent advances in the field of untargeted metabolomics have greatly facilitated the identification of compounds within root exudates. It can be expected to generate a deeper understanding of the composition and function of exudates (van Dam and Bouwmeester 2016).

The increasing occurrence of drought is predicted globally, which can hamper agricultural productivity (Dai 2011). Therefore, it is of utmost importance to understand the impact of drought stress on rhizosphere processes discussed above. Several studies have demonstrated the effect of drought stress on the growth and morphology of roots, including a shorter life span, deep rooting to fetch water from below ground, smaller xylem diameter and disruptions in root to shoot communications (Farooq et al. 2009; Comas et al. 2013). In contrast, the effect of drought stress on root exudation is poorly understood because of low accessibility and labour-intensive extraction methods (Aulakh et al. 2001; Strehmel et al. 2014; Gargallo-Garriga et al. 2018).

Pearl millet (*Pennisetum glaucum* (L.) R. Br. family: Poaceae, subfamily: Panicoideae) is an important multi-purpose cereal crop that provides food, fodder and fuel. It is evolved under the pressures of infertile soils, heat and drought, thus giving it a natural ability to thrive in low moisture, nutrient-deprived soils and at high temperatures, exceeding 40 °C (Varshney et al. 2017; Ghatak et al. 2017). It is widely cultivated throughout the arid and semi-arid regions of West Africa, East Africa and many parts of India (Oumar et al. 2008). The purpose of this study is to characterize root exudates of pearl millet and the effect of drought stress on their composition and concentration using high-performance liquid chromatography with ultraviolet detection (UV-HPLC), gas chromatography–mass spectrometry (GC–MS) and liquid chromatography–mass spectrometry (LC–MS). We also determined BNI activity mediated by root exudates of pearl millet after drought stress. We compared two different genotypes of pearl millet that originate from different geographic regions and are routinely used as high-yield staple food crops. Using this approach, we aimed to (1) identify physiological traits associated with a response to the deleterious effects of drought stress, (2) explore drought stress-related changes in the composition of root exudates in different genotypes and (3) assess the effect of drought stress on BNI activity.

## Materials and methods

### Plant samples, growth conditions, drought treatment and collection of root exudates

Two different pearl millet genotypes (sensitive (843-22B) and tolerant (ICTP8203) towards drought stress) from different states of India, primarily used for breeding, research and consumption, were used in the study. These are the high-yielding lines that are regularly used in the fields, the flowering time of these genotypes ranges between 65 and 67 days. We have obtained the seeds and information from the gene bank repository of the International Crops Research Institute for the Semi-Arid Tropics (ICRISAT), Patancheru, Hyderabad, India (http://genebank.icrisat.org/). These genotypes were evaluated for root exudate composition after drought stress considering four biological replicates in each condition (control and drought). This experiment was performed under controlled greenhouse conditions (12 h of light, max 30 °C during the daytime, 26 °C at night (± 2 °C), 60% relative air humidity and 80% at nighttime; the light was provided by metal halide lamps (HRI-TS 250 W/NDL Neutral white, Radium, Germany). The average PAR dosage was measured at 30 cm above pots and amounted to 600 μmol m^−2^ s^−1^ at noon. Growth and drought treatment was performed in custom-made cylindrical pipes according to Ghatak et al. (Ghatak et al. 2016, 2021) with a height of 75 cm (made up of 5 polyethylene segments (15 cm each)) and an inner diameter of 10.3 cm (Fig. S1). The total growth substrate volume was 6.25 L. Substrate moisture and substrate temperature were continuously monitored by means of 1% accurate theta probes (ADC ML3™) sensors. Each tube had two access openings for substrate moisture and substrate temperature monitoring by sensors, one in the upmost segment and one in the 2nd segment from the bottom. The growth substrate consisted of 3 parts of pot ground (peat, humus), two parts of quartz sand and 1 part of styromull. To avoid any leakage of the substrate and allow water to drain, the tubes had small perforations at the bottom. Drought stress was initiated with the development of the first panicle, and at this stage, plants were 56 days old. The difference in substrate moisture content between control and stressed plants was the first indication of the drought stress imposed, and significant physiological responses were measured (see below). Details of the genotypes, and their abbreviations used in the manuscript text, figures and tables have been provided in Table 1.

**Table 1:** Details of the genotypes and abbreviations used in the figures and tables

Cereal crop	Genotype name	Genotypes	Conditions applied	Abbreviations for figures and tables
Pearl millet (PM)	843-22B	Sensitive (S)	Control (C)	PM-S-C
			Stress (St)	PM-S-St
	ICTP 8203	Tolerant (T)	Control (C)	PM-T-C
			Stress (St)	PM-T-St

The effect of the drought treatment on the aboveground parts of the plants was examined by measuring chlorophyll fluorescence (F_v_/F_m_) using a plant efficiency analyzer (PEA) (Handy PEA, Hansatech Instruments, King’s Lynn, UK) in the morning (10:30 a.m. CET). Mature and fully expanded leaves were used for measurements in 4-day intervals throughout the entire experiment. The measurements were performed non-destructively on plant attached leaves. To quantify and compare physiological responses in the genotypes of pearl millet under control and stress conditions, parameters such as plant weight and root length were recorded after the treatment. For these measurements, the cylindrical pipes were dismantled, and the intact plants were removed from the substrate. Harvest index (HI) was calculated with the formula: harvest index (HI) % = [total yield (i.e. seed weight)/total yield + plant weight (i.e. biomass)] × 100.

After the drought treatment, roots were carefully removed from the substrate, cleaned thoroughly with deionized water and incubated in 500 mL distilled water. The root exudates were allowed to accumulate for 48 h at a lower temperature to primarily extract exudates from the root. After the incubation, the root exudate solution was filtered (0.2-μm pore size) to remove any root debris. Additionally, the samples were centrifuged at 20,000 g for 10 min at 4 °C (Subbarao et al. 2006a). Water was found to be the most effective accumulating solution for exudates (Valentinuzzi et al. 2015). Each 500 mL sample was concentrated to a volume of 10 mL using a rotary evaporator under vacuum at 40 °C. The concentrated root exudates were stored at 4 °C until further analysis. The dry weight of the roots was recorded according to Subbarao et al. (Subbarao et al. 2013a).

### UV-HPLC analysis of root exudates

For UV-HPLC analysis, 2 mL of the root exudates were further concentrated to 100 μL using speedvac (SCANVAC Cool Safe 110–4, Speed Vacuum concentrator, Labogene). The concentrated sample was dissolved in 400 μL water:methanol (MeOH):hydrochloric acid (HCl) solution (9:1:0.5, v/v/v), vortexed for 2 min and then centrifuged at 10,000* g* for 2 min at 4 °C. The supernatant was then transferred into HPLC micro vials with micro inserts and closed with crimp caps.

The HPLC system consists of a Dionex Summit equipped with a photodiode array detector (PDA) and attached to a FAMOS autosampler. A Phenomenex Synergi Max C12 column was used with the following dimensions: 150 mm × 2 mm, 4 μm particle size. The column oven was adjusted to 40 °C, and the flow rate was maintained at 0.2 mL min^−1^. Solvent A consists of water:MeOH:o-phosphoric acid (9:1:0.5, v/v/v); solvent B consists of pure MeOH . The pump system consists of a P680 HPLC pump. The gradient started at 100% of solvent A for 2 min and then linearly changed to 100% of solvent B within 98 min. The final concentration was held further for 20 min. The injection volume was 5 μL of the sample solution. UV spectra were recorded at 220 to 590 nm.

For HPLC data analysis, the Chromeleon software was used (Thermo Scientific). Retention time (RT), relative peak area, peak shape and peak height were measured using the library installed in the software, which contains reference peaks of measured standards and samples.

### GC–MS analysis of root exudates

Metabolite extraction was performed according to Weckwerth et al. (2004), with slight modifications. Two millilitres of root exudate solution was further concentrated to 200 μL using a speed vac (SCANVAC Cool Safe 110–4, Speed Vacuum concentrator, Labogene). The metabolites were extracted by addition of 750 μL pre-chilled extraction solution MeOH:water (MW) (2.5:1.5 v:v) to the concentrated exudates; the mixture was vortexed thoroughly, followed by incubation on ice for 15 min with periodic vortexing. After incubation, the samples were centrifuged at 20,000* g* for 4 min at 4 °C, and the supernatant was transferred to a new safe lock Eppendorf tube. The remaining pellet was washed once again with 250 μL of pre-chilled MW solution. The supernatant was pooled with the previously collected MW solution, 300 μL water was added and the samples were mixed and centrifuged for 2 min at 20,000* g* at 25 °C. The supernatant was collected, and the samples were dried using a speed vac (SCANVAC Cool Safe 110–4, Speed Vacuum concentrator, Labogene). All analysis steps, including sample derivatization and GC-TOF–MS (gas chromatography coupled to time-of-flight mass spectrometry), were carried out as previously described (Weckwerth et al. 2004; Obermeyer et al. 2013).

Data analysis was performed using the ChromaTOF (Leco) software. Chromatograms of different samples were used to generate a reference peak list, and all other data files were processed against this reference list. Retention index markers were used to calculate retention indices of compounds and for chromatographic alignment. Deconvoluted mass spectra were matched against an in-house mass spectral library, and the retention index was used for peak annotation. Peak annotations and peak integrations were checked manually before exporting peak areas for relative quantification into Microsoft Excel. Areas of different trimethylsilyl derivatives of single metabolites were summed, and from methoxyamine products, only one peak was selected for further analysis (De La Harpe et al. 2020; Pazhamala et al. 2020).

### LC–MS analysis of root exudates

For secondary metabolite extraction, 2 mL of the root exudates was concentrated to 100 μL using a speed vac (SCANVAC Cool Safe 110–4, Speed Vacuum concentrator, Labogene). For the RP-HPLC-HRESIMS (reverse-phase–high-performance liquid chromatography–high-resolution electrospray ionization mass spectrometry) measurements, the root exudates were dissolved to a concentration of 10 mg/L in a solvent consisting of (95% water, 5% acetonitrile (ACN) + 0.1% formic acid (FA)). Hundred microlitres of the supernatant were transferred into a glass insert for the analysis. The samples were placed into an autosampler at 4 °C. The stationary phase consisted of an Accucore™ Vanquish™ C-18^+^ UHPLC column (100 mm × 2.1 mm; 1.5-μm particle size). The mobile phase system consisted of a mixture of solvent A, an aqueous solution of 0.1% FA, and solvent B, ACN containing 0.1% FA. Ten microlitres of the sample was injected with a flow rate of 0.1 mL/min. The column compartment was kept at 30 °C. Between each measurement, a column washing step was performed by injecting a solution composed of 95% water, 5% ACN + 0.1% FA.

HRESIMS measurements were conducted using a HESI II (heated electrospray ionization) source in positive- and negative-ion mode connected to a Velos Pro, leading into a Thermo LTQ Orbitrap Elite instrument equipped with Xcalibur software (Thermo Scientific, Waltham, MA, USA). In positive-ion mode, the resolution was set at 120,000, a spray voltage of 4.5 kV was used and the capillary temperature was set at 300 °C. The mass scanning range of the MS 1 full scan was set at 100–1800 *m/z*. Nitrogen sheath gas and N auxiliary gas were set at a flow rate of 5 a.u and 2 a.u, respectively. Cyclomethicone N5 was used as a lock mass with an *m/z* value of 371.101230. Each MS 1 full scan was followed up by a maximum of 10 data-dependent MS 2 fragmentation spectra of the most abundant ion species. The time for dynamic exclusion duration of previously measured analytes was set at 30 s. The collision energy for CID was set at 35 eV. For measurements in negative-ion mode, the resolution was set at 120,000, a spray voltage of 3.5 kV was used and the capillary temperature was set at 350 °C. The mass scanning range of the MS 1 full scan was set at 100–1800* m/z*. N sheath gas and N auxiliary gas were set at a flow rate of 5 a.u. and 2 a.u, respectively. Each MS 1 full scan was followed up by a maximum of 10 data-dependent MS 2 fragmentation spectra, of the most abundant ion species. The time for dynamic exclusion duration of previously measured analytes was set at 30 s. The collision energy for CID was set at 35 eV. To reduce bias from measurement conditions, the samples were randomized and a blank arranged in a randomized order. Before the samples were measured, two measurements, each followed by a column washing step, were performed to ensure system equilibrium. A solution composed of (95% water, 5% ACN + 0.1% FA) was injected for all non-sample-type measurements.

The obtained Xcalibur RAW files were converted to the mzXML data format using MSConvert included in the ProteoWizard 3.0.9935 open-source software. For the conversion, mzXML was chosen as the output format with 32 bit as the binary encoding precision. The write index and trans-proteomic pipeline (TPP) compatibility boxes were marked. In the filters, section peak picking with Vendor algorithm was chosen for MS level 1–3. Peak picking, alignment and analyte annotation on MS 1 and MS 2 levels was performed by the in-house developed software mzFun using a standard database. The minimum peak height was set at 10,000 for MS 1 level. The minimum scan number was set at 5, and the S/N ratio was set at 3 for the untargeted searching and alignment.

### Annotation of LC–MS metabolites

Tentative annotation of metabolites was performed by manually screening the raw files for candidate metabolites by exact mass and comparing the MS2 fragmentation spectra with reference library data (mzCloud, MONA). *m*/*z* features that were not yet present in the reference database were also tentatively annotated by fragmentation pattern analysis. After annotation, peak integration was performed using the Thermo Xcalibur software. For the processing setup, each *m*/*z *feature was assigned an exact mass and expected retention time. The retention window was set at 600 s and the mass tolerance was set at 5 ppm. Peak detection was done using the ICIS algorithm. ICIS peak integration settings were set at 1 for smoothing points, 40 for baseline window, 5 for area noise factor and 10 for the peak noise factor. ICIS peak detection was set to the highest peak. The minimum peak height (S/N) was set to 3. The preliminary results were checked using Thermo Xcalibur Quanbrowser Software, and the peak integration was corrected manually if necessary. The resulting data matrix was reduced by only keeping *m*/*z* features present in at least 3 out of 4 replicates per condition.

### Biological nitrification inhibition (BNI) assay

BNI activity of all processed samples was determined using a nitrification inhibition bioassay that employs a strain of recombinant luminescent *Nitrosomonas europaea* (Subbarao et al. 2006a). Two millilitres of root exudate were completely dried using a speed vac. The dried exudates were dissolved in 200 μL of MeOH. The methanol extract was then evaporated to dryness using a rotary evaporator at 35 °C, and the resulting residue was extracted with 50 μL of dimethyl sulfoxide (DMSO); the DMSO extract was then used for the determination of BNI activity (Subbarao et al. 2006a). The total BNI activity of the samples is expressed in units defined in terms of the action of a standard inhibitor, allylthiourea units (ATU); the inhibitory effect of 0.22 μM AT in an assay containing 18.9 mM of NH_4_^+^is defined as one ATU (AT unit) of activity (Subbarao et al. 2006a, 2007b). Specific BNI activity is calculated based on BNI activity per gram root dry weight.

### Data processing and statistical analysis

Statistical analysis for physiological data points was performed using Statgraphics (ver. 17.2.05), and Umetrics SIMCA (version 13) was used for HPLC data to perform orthogonal projections to latent structures discriminant analysis (OPLS-DA). Data were centred and scaled by z-transformation. GC–MS and LC–MS data were subjected to multivariate analysis using the statistical toolbox COVAIN (Sun and Weckwerth 2012). Box plots and bar graphs were constructed using R (version 3.5, R Core Team 2019) (package ggplot2).

## Results

### Physiological traits demonstrate genotype-specific acclimatization strategies under drought stress

**Fig. 1:**
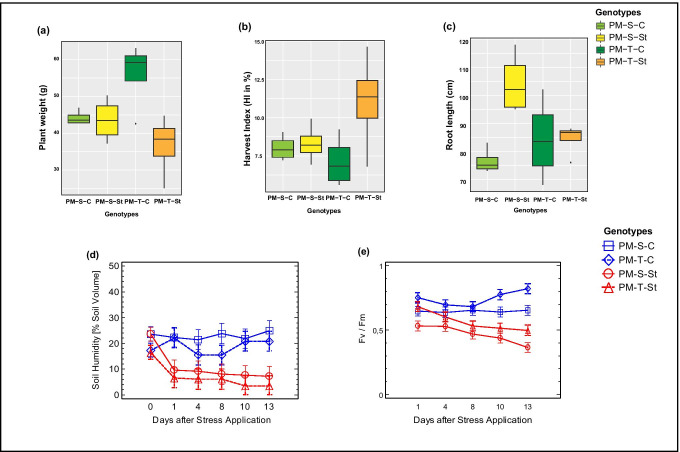
Physiological parameters measured under control and drought stress conditions in pearl millet genotypes (843-22B and ICTP8203) (*n:4*). **a** Box plot represents plant weight (g); **b** box plot represents harvest index (HI) in percentage; **c** box plot represents root length (cm); **d** substrate moisture was measured using sensors ML 3 ThetaProbe; **e** chlorophyll fluorescence (F_v_/F_m_) determined using plant efficiency analyzer (PEA)

In order to investigate the genotype-dependent physiological variation under drought stress, parameters such as plant weight, root length, harvest index and F_v_/F_m_ were determined. Substrate moisture content was continuously monitored using sensors (ADC ML3™) revealing specific physiological adaptations of the compared genotypes (Fig. 1). Physiological data attributed to growth parameters were affected significantly and differently between the compared genotypes (sensitive 843-22B and tolerant ICTP8203). Overall, plant weight was reduced under drought stress in both the genotypes; a significant reduction was observed in ICTP8203 (Fig. 1a). The harvest index showed a strong genotype-dependent response to drought stress due to contrasting plant weight reduction (Fig. 1b). Root length also varied strongly under drought stress pointing to genotype-specific responses (Fig. 1c). 843-22B showed highly elevated root length growth under stress conditions in contrast to ICTP 8203 (Fig. 1c).

Drought stress reduced the maximum efficiency of PSII photochemistry (F_v_/F_m_); however, this effect varied in its severity among the compared genotypes (Fig. 1e). By the end of the drought regime, Fv/Fm ratio was more decreased in the sensitive variety 843-22B compared to ICTP8203.

### Root exudate profiling using HPLC–UV analysis

**Fig. 2:**
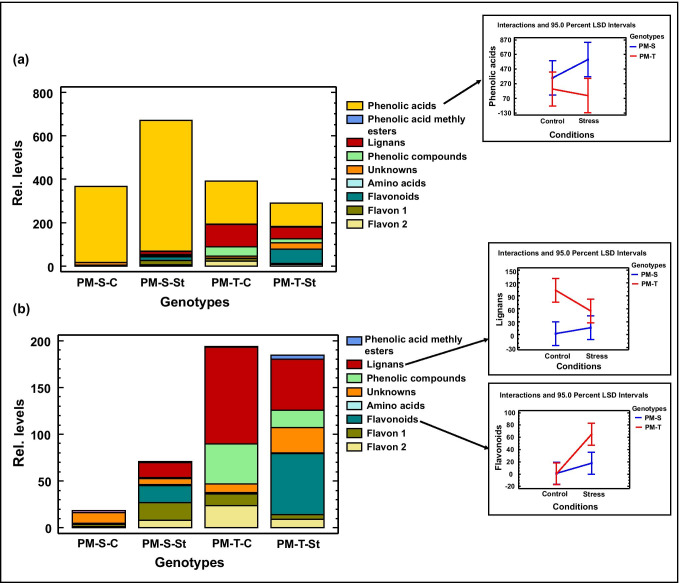
UV-HPLC results: **a** relative abundance of the compounds identified in root exudate including phenolic acids; **b** relative abundance of the compounds identified in root exudate excluding phenolic acids (*n:4*, error bar = confidence interval 95%)

Using HPLC–UV analysis 97 compounds were detected and quantified. The compounds were classified into nine different categories: phenolic acid, phenolic acid methyl esters, lignans, phenolic compounds, amino acids, flavonoids, flavon class 1 and class 2 and unknown compounds (Table S1; Fig. 2a, b). Drought stress significantly altered root exudate composition in these metabolite profiles in both genotypes. OPLS-DA analysis revealed the separation between the compared genotypes (843-22B and ICTP8203) and also the stress treatments (Fig. S2). The strongest variation between samples could be observed between control and stressed samples of ICTP8203, the tolerant genotype on component 1.

An enhanced concentration of phenolic acids and lignans was observed under stress conditions in 843-22B, while opposite regulation was observed in ICTP8203 (Fig. 2a, b). Interestingly, flavonoids were not detected under the well-watered condition of ICTP8203 but showed higher concentration after a drought stress period (Fig. 2b).

### Genotype-specific changes in the exudation of primary metabolites measured by GC–MS analysis

Untargeted metabolite analysis was performed using gas chromatography coupled to mass spectrometry (GC–MS). Peaks were identified by analysis of retention index (RI) and mass spectra (Ghatak et al. 2018). In total, 49 compounds were identified at level 1 (Goodacre et al. 2007) and quantified. These can be broadly classified into amino acids, sugars, organic acids and other compounds (Table S2). The principal component analysis (PCA) determined the strongest variation between control and stress samples in 843-22B on principal component 1 (Fig. S3). The loadings of principal component 1 (PC1) revealed a unique set of metabolites, which showed enhanced accumulation under stress conditions (Table S3). The positive loadings include metabolites such as mevalonic acid, malic acid, carbohydrates and other organic acids. Interestingly, in ICTP8203, a very week separation was observed between control and stress conditions (Fig. S3, Table S3).

### Profiling of secondary metabolites in root exudation using LC–MS analysis

Liquid chromatography–mass spectrometry (LC–MS) metabolite profiling led to the identification of 332 *m/z* features from positive-ion mode and 958 *m/z* features from negative-ion mode by the in-house developed software mzFun (Table S4). Principal component analysis of both positive and negative modes showed a clear separation between the compared genotypes under stress conditions (Fig. S4 a, b). Furthermore, 17 compounds were annotated using manual curation of positive-ion mode measurements (Table 2). The level of identification was assigned according to Sumner et al. (2007). These compounds contribute to the separation of the compared genotypes under stress conditions. Fragmentation spectra and regulation of these compounds in the compared genotypes under control and stress conditions are visualized in Fig. S5.

**Table 2:** List of annotated compounds detected by LTQ Orbitrap Elite. Level of identification was defined according to Sumner et al. (2007)

No. of compounds	Name of compounds	Level of identification notes	Level of identification	Formula	Ion Species	*m/z*	RT (min)	Mass accuracy (in ppm)
1	Acetyl carnitine	MS2	2	C_9_H_17_NO_4_	[M + H]^+^	204.123	2.80	0.00
2	Adenosine	MS2	2	C_10_H_13_N_5_O_4_	[M + H]^+^	268.104	3.33	0.00
3	Guanosine	MS2	2	C_10_H_13_N_5_O_5_	[M + H]^+^	284.0989	3.62	0.00
4	Leucine/isoleucine	MS2	2	C_6_H_13_NO_2_	[M + H]^+^	132.1019	4.26	1.51
5	Indoleacetic acid	MS2	2	C_10_H_9_NO_2_	[M + H]^+^	176.0706	21.00	− 1.14
6	Genistein/galangin	MS2	2	C_15_H_10_O_5_	[M + H]^+^	271.0601	26.39	0.74
7	Lysine	MS1	3	C_6_H_14_N_2_O_2_	[M + H]^+^	147.1128	4.26	2.04
8	Pantothenic acid	MS2	2	C_9_H_17_NO_5_	[M + H]^+^	220.118	7.02	− 1.36
9	Riboflavin	MS2	2	C_17_H_20_N_4_O_6_	[M + H]^+^	377.1456	14.98	− 1.86
10	Unknown 4 Putative phenolic hexosyl rhamnoside dimer	MS2	3	C_52_H_64_O_26_	[M + H]^+^	1105.3759	19.22	− 1.00
11	Unknown 2 Putative phenolic hexosyl rhamnoside	MS2	3	C_26_H_32_O_13_	[M + H]^+^	553.1916	19.90	− 0.90
12	Unknown 5 Putative phenolic hexoside	MS2	3	C_20_H_22_O_9_	[M + H]^+^	407.1337	19.31	− 1.47
13	Unknown 6 Putative phenolic hexoside dimer	MS2	3	C_40_H_44_O_18_	[M + H]^+^	813.26	19.45	− 1.23
14	Unknown 3 Putative phenolic hexosyl rhamnoside	MS2	3	C_26_H_32_O_13_	[M + H]^+^	553.1916	19.22	-1.27
15	Unknown 7 Putative phenolic dimer	MS2	3	C_28_H_24_O_8_	[M + H]^+^	489.1544	25.54	− 1.84
16	Unknown 1 Putative phenolic	MS2	3	C_14_H_12_O_4_	[M + H]^+^	245.0808	26.06	− 1.63
17	Unknownn 8 Putative phenolic dimer	MS2	3	C_28_H_24_O_8_	[M + H]^+^	489.1544	26.23	− 2.04

### Effect of drought stress-induced root exudates on nitrification

**Fig. 3:**
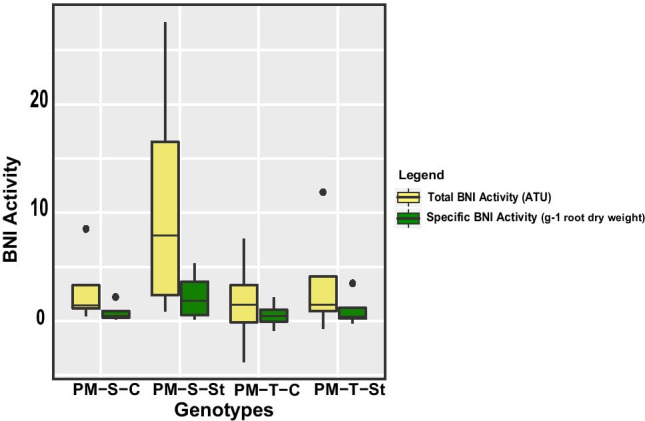
Biological nitrification inhibition (BNI) activity (*n:4*): total BNI activity and specific BNI activity were measured in the root exudates of pearl millet under control and drought stress

Root exudates collected from the pearl millet genotypes after a drought stress period showed a wide range of BNI activity (i.e. the ability to release BNI compounds from roots) (Fig. 3). Total BNI activity was detected in the range of 1.71 to 11.04 allylthiourea units (ATU), whereas the specific BNI capacity was found to be in the range of 0.56 to 2.31ATU g^-1^ root dry weight in both the genotypes under control and stress. Exposing the roots to drought stress resulted in higher total BNI activity and potential BNI compounds released per unit root mass. 843-22B under drought stress showed the highest BNI activity (11.04 ATU total BNI) and accounted for 2.31 ATU g^−1^ root dry weight-specific BNI, compared to ICTP8203 where the BNI capacity was measured to be 3.54 ATU total BNI and 1.03 ATU g^−1^ root dry weight-specific BNI (Fig. 3).

## Discussion

The effect of drought on the physiology and the regulation of metabolites present in root exudates has not been investigated extensively. However, it can be of importance in climate change scenarios where aridity is increasing in several areas of the globe. Furthermore, root exudates have the ability to manipulate the soil microbiome composition and biochemical activities, especially nitrification activity by biological nitrification inhibition (BNI), to improve the N use efficiency of the plant (Nardi et al. 2020; Subbarao and Searchinger 2021). There is not much knowledge of how stress affects root exudation and plant–soil interactions. We have addressed this question in the present study by applying drought stress and further investigating the potential of different pearl millet varieties with respect to BNI activity. We investigated two significantly different varieties, a drought-sensitive genotype 843–22B and a drought-tolerant genotype ICTP 8203. Drought stress affected the physiological traits (such as plant weight, root length, harvest index and F_v_/F_m_) and initiated a genotype-specific response (Fig. 1). The total plant weight of the plants was reduced significantly in the tolerant genotype ICTP 8203 compared to the sensitive genotype 843–22B but at the same time ICTP 8203 showed the highest harvest index (Fig. 1). This response from the drought-tolerant genotype can be explained as an adaptive response (Ghatak et al. 2021). Plants maintain low tissue water content via osmotic adjustment and cellular plasticity to divert their entire energy to protect seed productivity under stress conditions (Farooq et al. 2009). Pearl millet is generally drought tolerant and exhibits true stress tolerance mechanisms because a very low amount of water is required to sustain growth and yield. Pearl millet genotypes can acquire water from a deep soil layer (50 cm and below) (Kusaka et al. 2005). As drought stress is directly coupled to N deficiency (Waraich et al. 2011) and because of lower uptake rates of minerals, this may be an alley to pursue further. The drought-induced root elongation of the sensitive genotype 843-22B (Fig. 1) is an indication of such causality and at the same time brings up the hypothesis of altered root metabolism and exudation.

HPLC–UV characterization of the root exudates demonstrated significant alteration in both genotypes of pearl millet (843-22B and ICTP 8203) after drought stress. Compounds such as phenolic acids, lignans and flavonoids were significantly affected under drought stress and demonstrated genotype-specific response (Fig. 2a, b). Phenolic acids may be the response to abiotic stress due to their oxidizability and reactive oxygen species (ROS) scavenging capacity (Chobot et al. 2016; Nakayama and Uno 2015). Lignans are the major component of the vascular plant cell wall, providing mechanical support. Under drought stress, they play an important role in the thickening and tightening of xylem tracheids, partly by the enhanced lignification of cell wall polymers (Brunner et al. 2015). Hence, lignans might play a very important role in root elongation of 843-22B under stress conditions.

Interestingly, the regulation of flavonoids was only enhanced under stress conditions in ICTP 8203. Flavonoids can function as antioxidants and are metabolites that accumulate under stressful environmental conditions. They play a direct role in drought tolerance and ROS scavenging. The increased levels of flavonoids likely play an important role in the defence and also signalling mechanisms (Steinkellner et al. 2007).

### Drought stress alters the exudation composition of primary metabolites

**Fig. 4:**
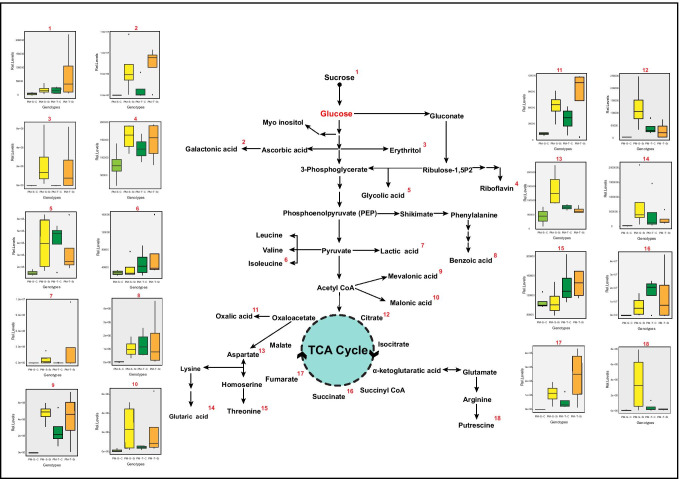
Regulation of the primary metabolites in the root exudates identified using GC–MS approach under control and drought stress condition in pearl millet genotypes (843-22B and ICTP8203) (*n:4*)

Metabolome characterization of root exudates under drought stress exhibited a strong effect on the concentration and composition of the exudates in the sensitive genotype (843-22B) as compared to the tolerant genotype (ICTP8203) (Fig. S3). The responses of the individual metabolites under control and drought stress in 843-22B and ICTP8203 are summarized in metabolic pathways in Fig. 4. Drought treatment enhanced the concentration of organic acids (succinic acid, oxalic acid, lactic acid, fumaric acid, malonic acid and citric acid) in both the compared genotypes. Increased accumulation of these compounds in the exudate of drought-stressed plants can promote osmotic adjustment which may help in maintaining root development to access water from deep layers of soil (Serraj and Sinclair 2002). Increased secretion of oxalic acid under stress conditions can protect roots against the damages inflicted by reactive oxygen species (ROS) resulting from interactions with the allelochemical substances (Weir et al. 2004). This response can be well corroborated with the growth response of 843-22B, which demonstrated enhanced root length under stress conditions. Root morphology such as root length plays an important role in shaping post-drought stress response and can vary across different species.

Furthermore, high accumulation of citrate under stress can be a prevalent response to nutrient deficiency in a dry environment (e.g. chelation, cleaving of carbonates) (Fig. 4). Interestingly, water-soluble vitamin such as riboflavin (vitamin B2) was also identified with higher accumulation under stress condition in both 843-22B and ICTP8203 (Fig. 4). Riboflavin is primarily important as a precursor of flavin mononucleotide (FMN) and flavin adenine dinucleotide (FAD); both are redox cofactors. Riboflavin can stimulate antioxidant production under drought stress conditions. In tobacco and *Agaricus bisporus* plants, riboflavin increased drought tolerance (Deng et al. 2014; Guhr et al. 2017).

### Altered regulation of secondary metabolites, vitamins and phytohormones determines genotype- and drought-specific responses

**Fig. 5:**
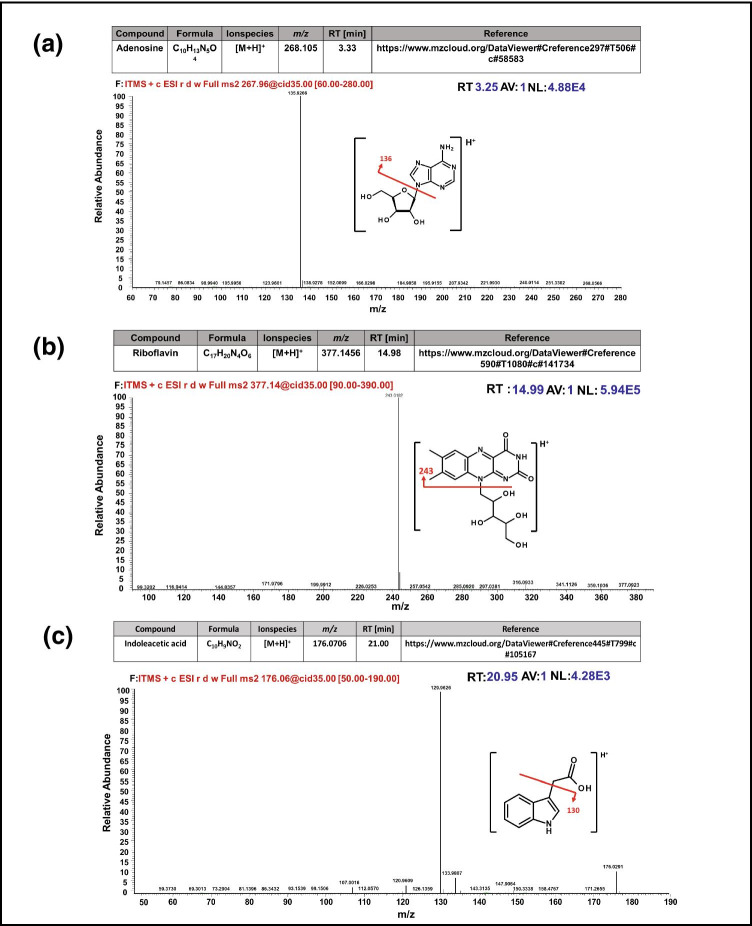
Fragmentation spectra of the secondary metabolite compounds identified in the root exudates using the LC–MS approach. The compounds were manually annotated **a** adenosine, **b** Riboflavin, **c** indoleacetic acid (IAA)

The differential regulation of secondary metabolites (Table 2) demonstrated a genotype-specific response under control and stress conditions (Fig. S5). Adenosine plays an important role in plant growth and development. A higher accumulation of adenosine was observed under drought stress in both genotypes. Adenosine can trigger protective mechanisms in plants under drought stress (e.g. defence mechanism against ROS) (Fig. 5a) (Macková et al. 2013; Xu et al. 2017). A similar response was observed in holm oak under drought stress (Gargallo-Garriga et al. 2018). Furthermore, we also identified water-soluble vitamins such as riboflavin and pantothenic acid, showing enhanced accumulation under stress conditions in both genotypes (Fig. 5b, c) (Schonwitz and Ziegler 1982). Higher accumulation of vitamins not only promotes the growth and development of plants but also controls the growth and composition of rhizosphere microflora. Riboflavin was also detected via GC–MS analysis with a similar regulation pattern. Phytohormones are the endogenous factors that modulate root system architecture (Bais et al. 2006). They also play an important role in the mitigation of abiotic stresses (Teale et al. 2008). In the present study, a higher accumulation of indole-3-acetic acid (IAA) was observed under stress conditions in both genotypes compared to controls.

Interestingly, several unknown phenolic compounds were also identified with enhanced accumulation under stress conditions in both genotypes (Table 1; Fig. S5). These compounds seem to be related to each other because they all contain a similar aglyconic part with the formula C_14_H_12_O_4_. This formula could be attributed to compound classes like stilbenoids, naphthoquinones, antraquinones and phenanthrenoids. Besides the aglycon and glycosylated varieties, dimeric forms of this compound class were also detected. Stilbenoids, naphthoquinones, antraquinones and phenanthrenoids are all reported as constituents of root exudates for various plants . Besides their anti-fungal and nematicidal properties, these compound classes were also shown to be involved in plant–microbe signalling interactions (Munakata et al. 2019). Since these compounds seem to be a major constituent of the pearl millet root exudate under drought stress, further investigation of isolation, structural elucidation and BNI activity potential could lead to a deeper understanding of plant drought stress responses and plant–microbe interactions.

### Assessment of BNI activity mediated by root exudates from two different genotypes of pearl millet

The BNI activity has been mainly explained by the release of biologically active compounds from roots that suppress or regulate the growth and function of the nitrifiers, which limits the nitrification process in soil (Coskun et al. 2017b; Nardi et al. 2020; Subbarao et al. 2006b; Subbarao and Searchinger 2021). This study provides the first evidence of genotypic differences in the BNI capacity of pearl millet where BNI release is substantially influenced by drought stress, showing enhanced activity in 843-22B as compared to ICTP8203 (Fig. 3). Plants under drought stress are subject to microclimatic changes in their soil habitat, i.e. soil water content is decreased. As a consequence, partial pressure of oxygen and the redox potential in the soil are increased. These changes accelerate nitrification and decelerate denitrification by the enzymatic activity of microorganism (BNI) or simple chemical redox processes in the soil. By exuding oxidizable phenolic compounds via their roots, plants can buffer these oxidation processes and protect the vital ammonium resource. Also, the root exudates may directly supply substrate to modulate associated microorganisms. This has been shown for maize (Mehmood et al. 2018), and we put forward a similar ecological functionality for *Pennisetum* root exudation. The compatible solute exudation (sugar alcohols, proline, quaternary ammonium compounds) will probably also protect the remineralizing activity of associated bacteria and archaea from losing their potential activity due to dehydration.

Based on our results, 843-22B can be considered an interesting model to study the BNI phenomenon due to its high BNI capacity. Furthermore, at the physiological level, root length of 843–22B was increased under stress conditions which can have a possible impact on the BNI activity because high levels of root exudation are associated with higher root length (Guyonnetet al. 2018). Most of the studies consider that the BNI phenomenon is mostly caused by the BNI activity release from exudation (Subbarao et al. 2006a, 2007a, 2009). However, recently a study demonstrated that the BNI released from root tissue of *Brachiaria* sp. also had a significant contribution to the inhibitory effect (Nakamura et al. 2020). Therefore, further studies are needed to understand the high BNI potential of root tissue independent of the inhibition caused by exudates.

### A conceptual model describing key system parameters of the plant–soil–microbiome interaction of two different pearl millet genotypes

**Fig. 6:**
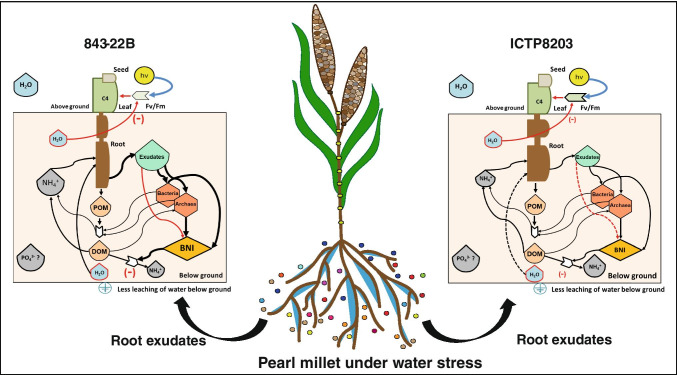
Mechanistic model describing modification of the key system parameters by the exudation process in the different genotypes of pearl millet (843-22B and ICTP 8203) under drought stress using the visualization approach by Odum. The proposed model visualizes different parts of the plants (i.e. seed, shoot and root system) and soil microbes consisting of bacteria and archaea. The key pool components in the models include water resources, ammonium availability, DOM, POM, root exudates and nitrate. Abbreviations: NH_4_^+^, ammonium availability; DOM, dissolved organic matter; POM, particulate organic matter; BNI, biological nitrification inhibition; H_2_O, water availability; PO4^3−^—phosphate availability; hv, light energy

In Fig. 6, we propose a working model for future investigations for both the genotypes of pearl millet under drought stress based on physiological responses, root exudates and the BNI activity (Fig. 6). The proposed model predicts belowground modification by the exudates of both the genotypes to generate different adaptation mechanisms to survive under drought stress. To integrate all the physiological effects of the plants, metabolome of the root exudates and BNI activity into an intuitive model, we used the system ecology visualization strategy by Howard Odum, a theoretical approach based on the integration of systems theory in ecology (Odum 1994). Here, each symbol and size determines a system state variable representative for the individual pearl millet genotypes under drought stress (Fig. 6). The key processes described in the model include rhizodeposition which in turn comprise of particulate organic matter (POM) and dissolved organic matter (DOM) low molecular weight organic compounds (LMWOC) fractions, mainly amino acids, carbohydrates, organic acids and BNI quenching substances (e.g. flavonoids), also the leaching of the water to below ground system and the impact of limited water availability on photosynthetic primary processes depicted in the form of the F_v_/F_m_ ratio.

Under drought stress, 843-22B demonstrated enhanced elongated but thinner roots and high aboveground biomass with reduced harvest index because the plants were not able to alter shoot to seed weight. Enhanced BNI activity suggests the modulation of plant–microbe interactions under drought stress. Consequently, we hypothesize that ammonium availability for plants may also be optimized by this activity but this needs further investigation. The enhanced exudation of LMWOC substrates that might be utilized by the soil microbes is able to modulate the plant–microbe interaction. Another fraction of exudates (e.g. flavonoids) directly inhibits ammonium oxidation (Subbarao et al. 2013a). These exudates qualify further as antioxidants, which are protecting ammonium compounds from oxidation, e.g. by ROS in the rhizosphere. The proposed model does not elaborate on phosphate availability which is a direction to pursue in future studies. The only hint of enhanced phosphate availability observed was an increase of organic acids among the exudates. In contrast, ICTP8203 exhibits no such adaptation mechanisms but in contrast revealed drought-tolerant behaviour. The shortage of water did not induce any belowground modification. In order to preserve productivity, the shoot biomass was reduced which is possible if sufficient air humidity does not impose severe water deficit pressure on the leaves (Fig. 6). Here, we hypothesize that limited air humidity may render the adaptation mechanisms of 843-22B and could stretch the tolerance of ICTP8203 to its limits. Hence, both the genotypes may be less suitable crops for harsher arid regions with low air humidity. This is an area of research which needs more consideration and experimental confirmation.

## Conclusions

This study is the first to document the changes in the root exudate composition of primary and secondary metabolites in the different genotypes of pearl millet after drought stress. The exudation composition significantly depends on the changes in water availability and subsequent alteration of root growth. An effect was also observed on the BNI capacity of the pearl millet plants, where 843-22B showed maximum BNI activity under drought stress. It is observed that the plant root length may not only be related to N deficiency, but also to enhance root exudation under drought stress, thereby initiating both avoidance and adaptation mechanisms. In direct comparison to 843-22B, these effects were less pronounced in ICTP8203. However, ICTP8203 may survive more arid conditions where the adaptation options for 843-22B reach their limits. In order to understand the composition of the metabolites within the root exudates of pearl millet, we used different analytical platforms such as HPLC–UV, GC–MS and LC–MS for characterization.

The results highlight putative marker compounds (e.g. flavonoids and ROS scavenging metabolites), which can be further evaluated for nitrification inhibition. Evaluation of these natural compounds can lead to a deeper understanding of BNI mechanisms which can translate into elite lines with higher natural BNI activity under drought stress and consequently a better nitrogen use efficiency (NUE).

Furthermore, the systematic application of multiomics in collecting and analyzing root exudates will provide a deeper understanding of the belowground plant–soil interactions under abiotic stress in order to enhance plant productivity and quality (Weckwerth et al. 2020).

## Supplementary Information

Below is the link to the electronic supplementary material.

Supplementary file1 Fig. S1: Pictures of the experimental setup in the glasshouse.Fig. S2: (a) Orthogonal partial least squares discriminant analysis (OPLS – DA) was performed considering all the compounds identified using UV-HPLC under control and stress condition in pearl millet genotypes (843-22B and ICTP8203). (b) Plot representing loadings of principal component analysis.Fig. S3: (a) Principal component analysis (PCA) of all the primary metabolites identified in the exudates of pearl millet genotypes (843-22B and ICTP 8203).Fig. S4: Principal component analysis (PCA) of the *m*/*z* features identified using in-house software mzFun (a) PCA of the *m*/*z* features identified from positive ion mode measurements (b) PCA of the *m*/*z *features identified from negative ion mode measurements.Fig. S5: Fragmentation spectra of the 17 secondary metabolites manually annotated. (PDF 1141 KB)

Supplementary file2 (XLSX 12 KB)

Supplementary file3 (XLSX 20 KB)

Supplementary file4 (XLSX 20 KB)

Supplementary file5 (XLSX 194 KB)
