# Supplementary material for: Root exudation of contrasting drought-stressed pearl millet genotypes conveys varying biological nitrification inhibition (BNI) activity
Source: Biol Fertil Soils. 2021 Jul 9;58(3):291–306. doi: 10.1007/s00374-021-01578-w (PMC8938368; doi:10.1007/s00374-021-01578-w)

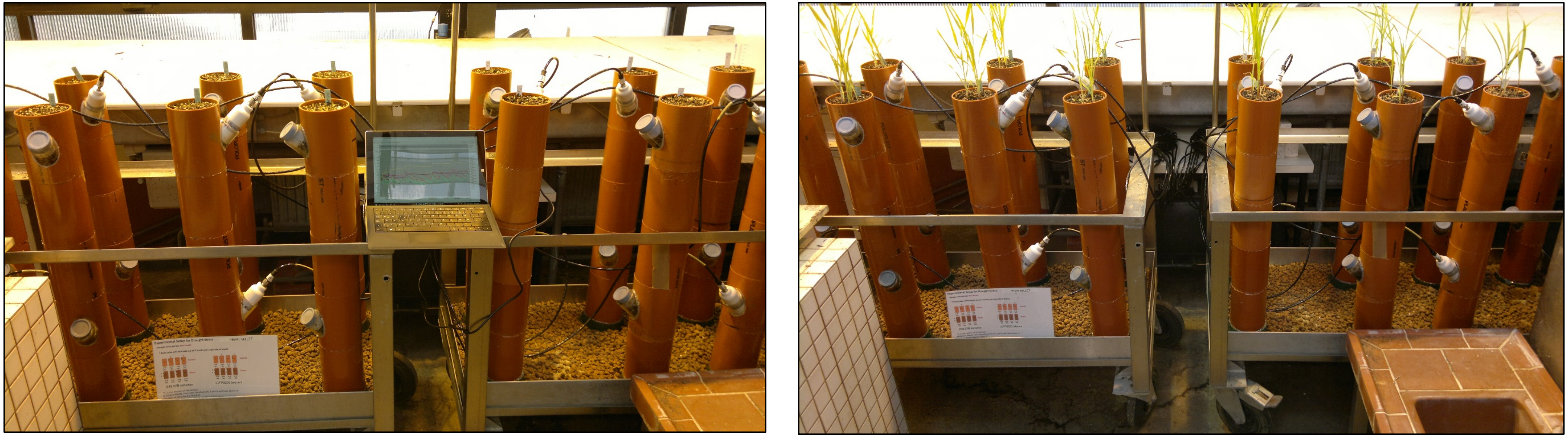

Fig. S1

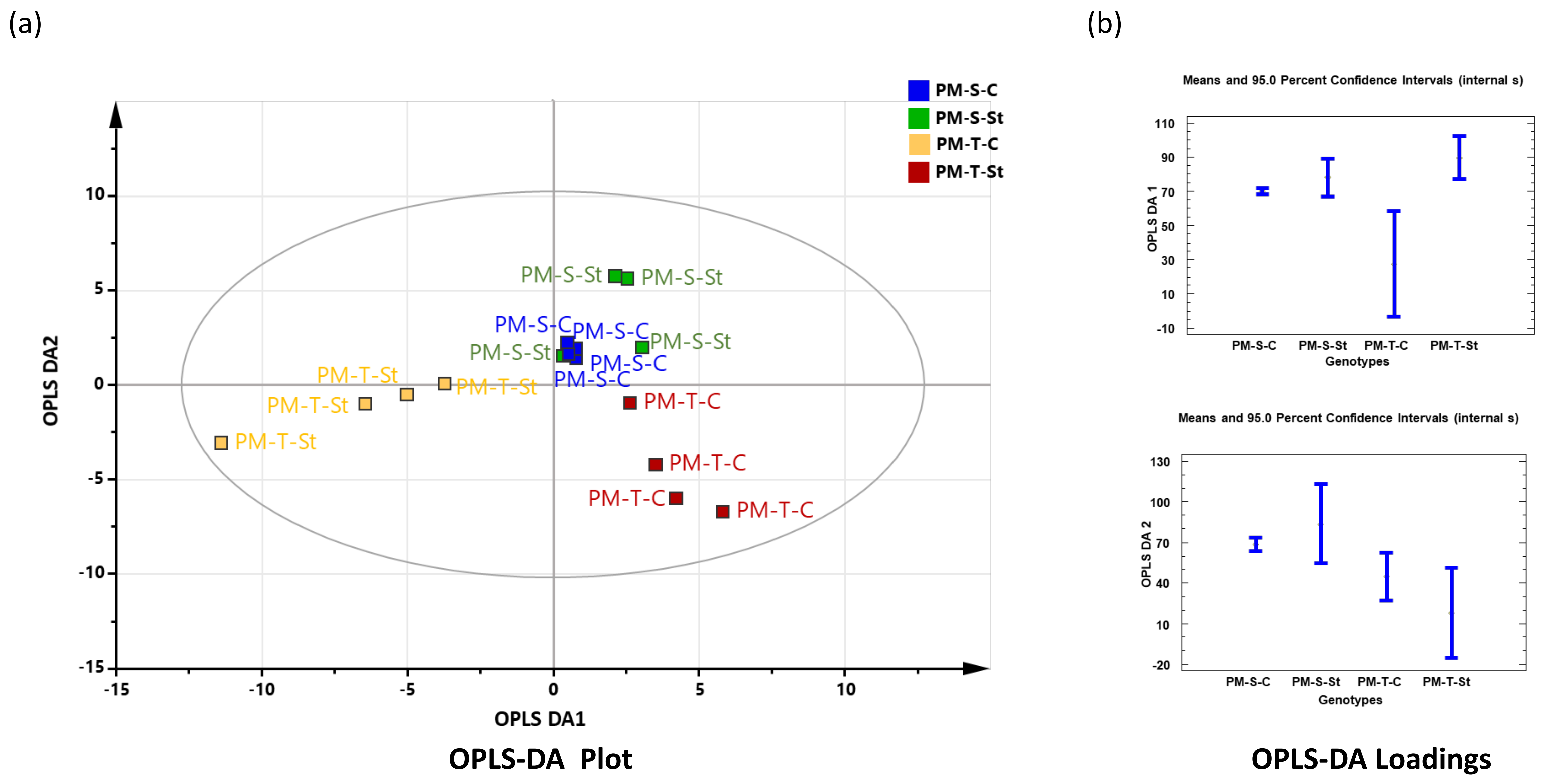

(a)
PM-S-C
PM-S-St
PM-T-C
PM-T-St
OPLS DA2
OPLS DA1
OPLS-DA Plot
(b)
Means and 95.0 Percent Confidence Intervals (internal s)
OPLS DA 1
Genotypes
Means and 95.0 Percent Confidence Intervals (internal s)
OPLS DA 2
Genotypes
OPLS-DA Loadings

Fig. S2

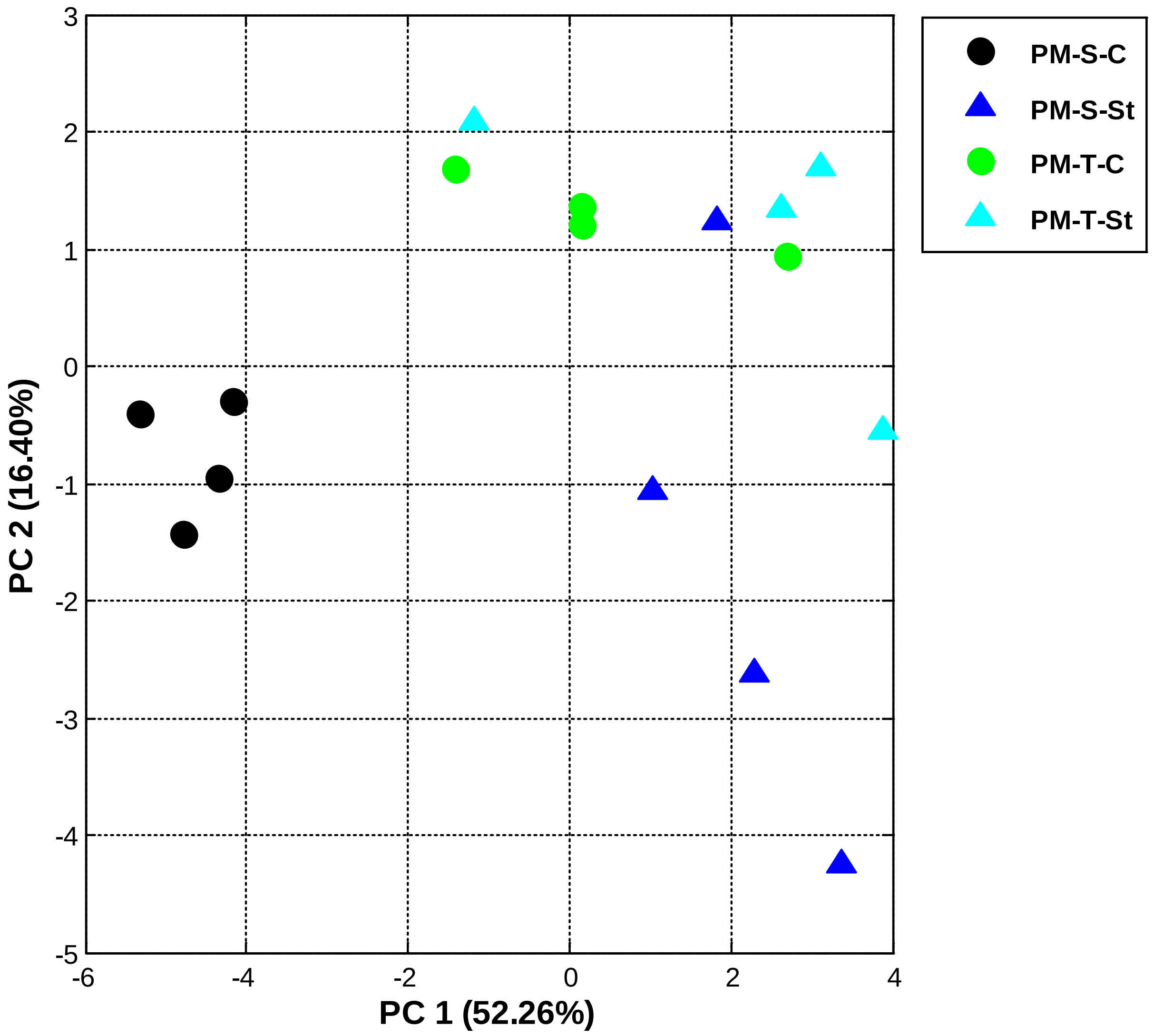

Fig. S3

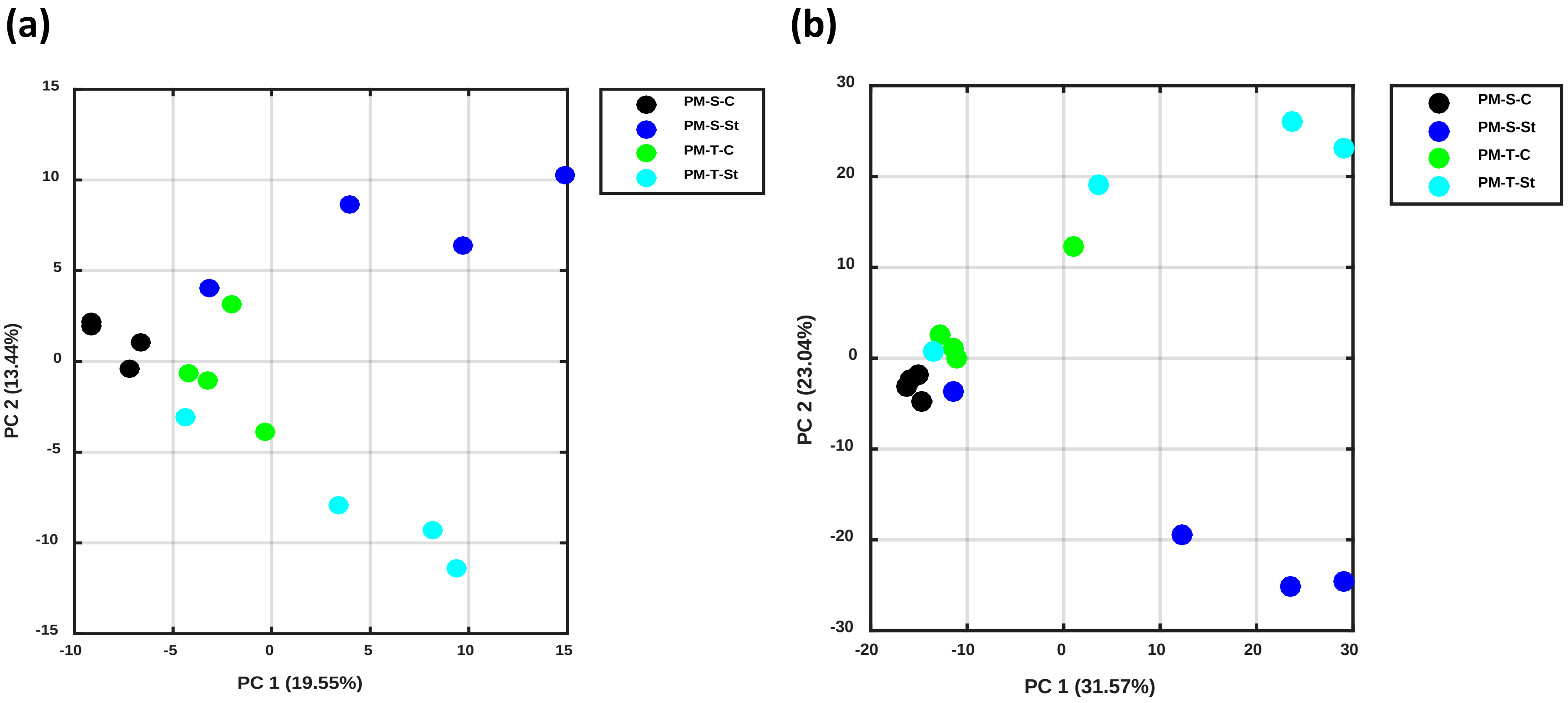

Positive mode: Untargeted Analysis

Negative mode: Untargeted Analysis

Fig. S4

Compound 1	Formula	Ionspecies	*m/z*	RT [min]	Reference
Adenosine	$C_{10}H_{13}N_5O_4$	$[M+H]^+$	268.105	3.33	https://www.mzcloud.org/DataViewer#Creference297#T506#c#58583

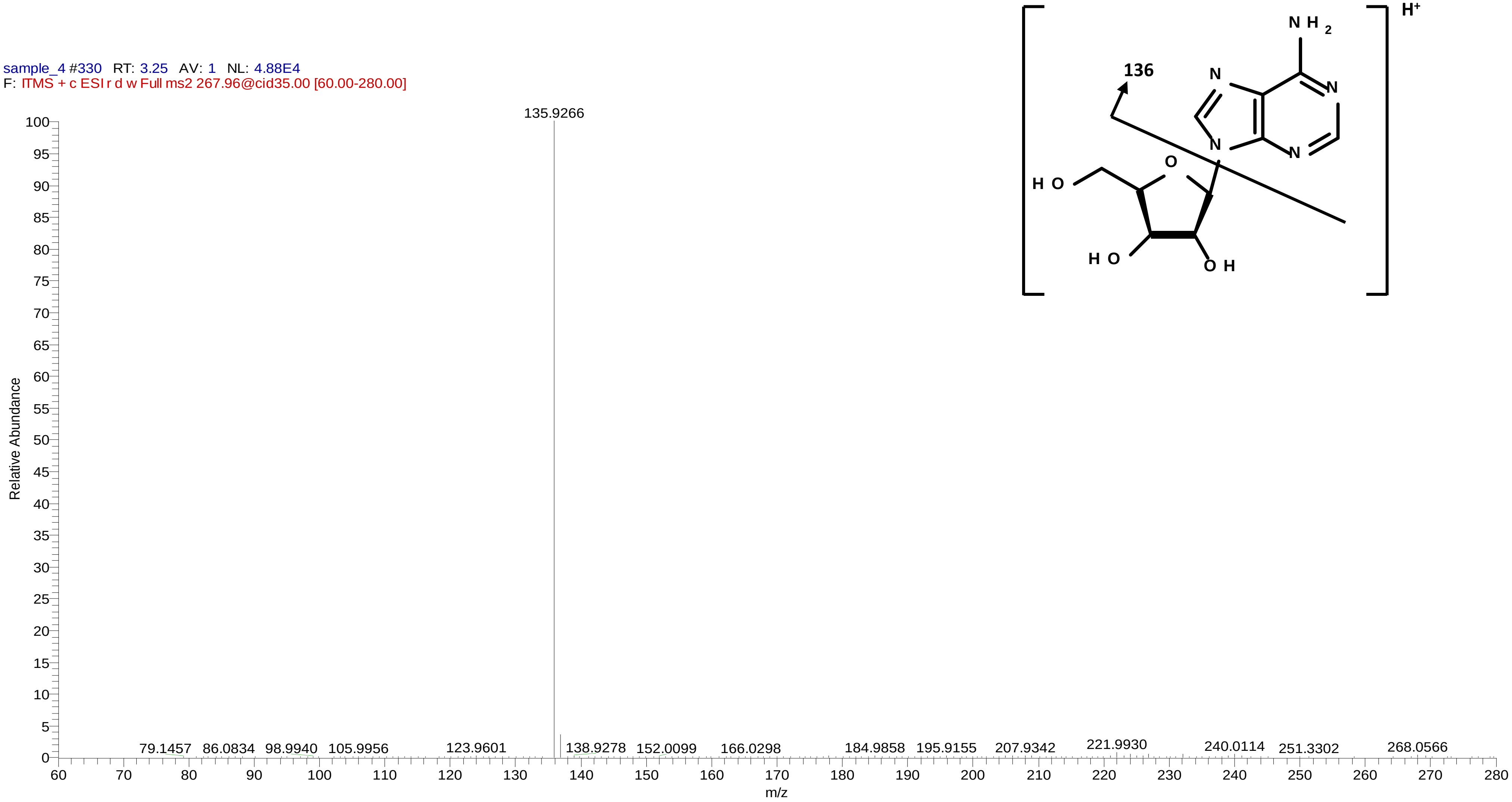

Fig. S5

Compound 2	Formula	Ionspecies	*m/z*	RT [min]	Reference
Guanosine	$C_{10}H_{13}N_5O_5$	$[M+H]^+$	284.0989	3.62	http://www.massbank.jp/RecordDisplay.jsp?id=KO008966&dsn=Keio_Univ

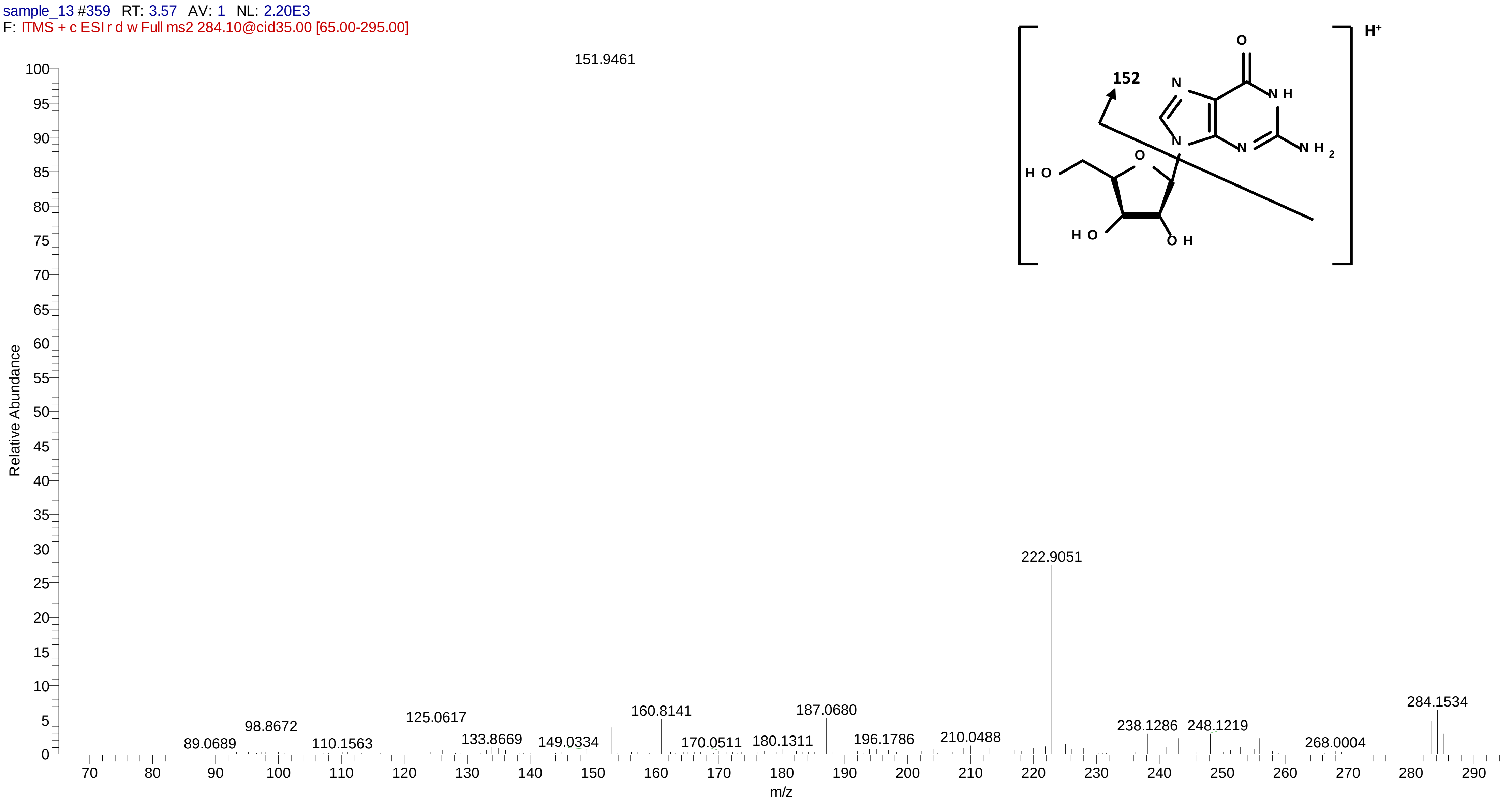

Compound 3	Formula	Ionspecies	*m/z*	RT [min]	Reference
Riboflavin	$C_{17}H_{20}N_4O_6$	$[M+H]^+$	377.1456	14.98	https://www.mzcloud.org/DataViewer#Creference590#T1080#c#141734

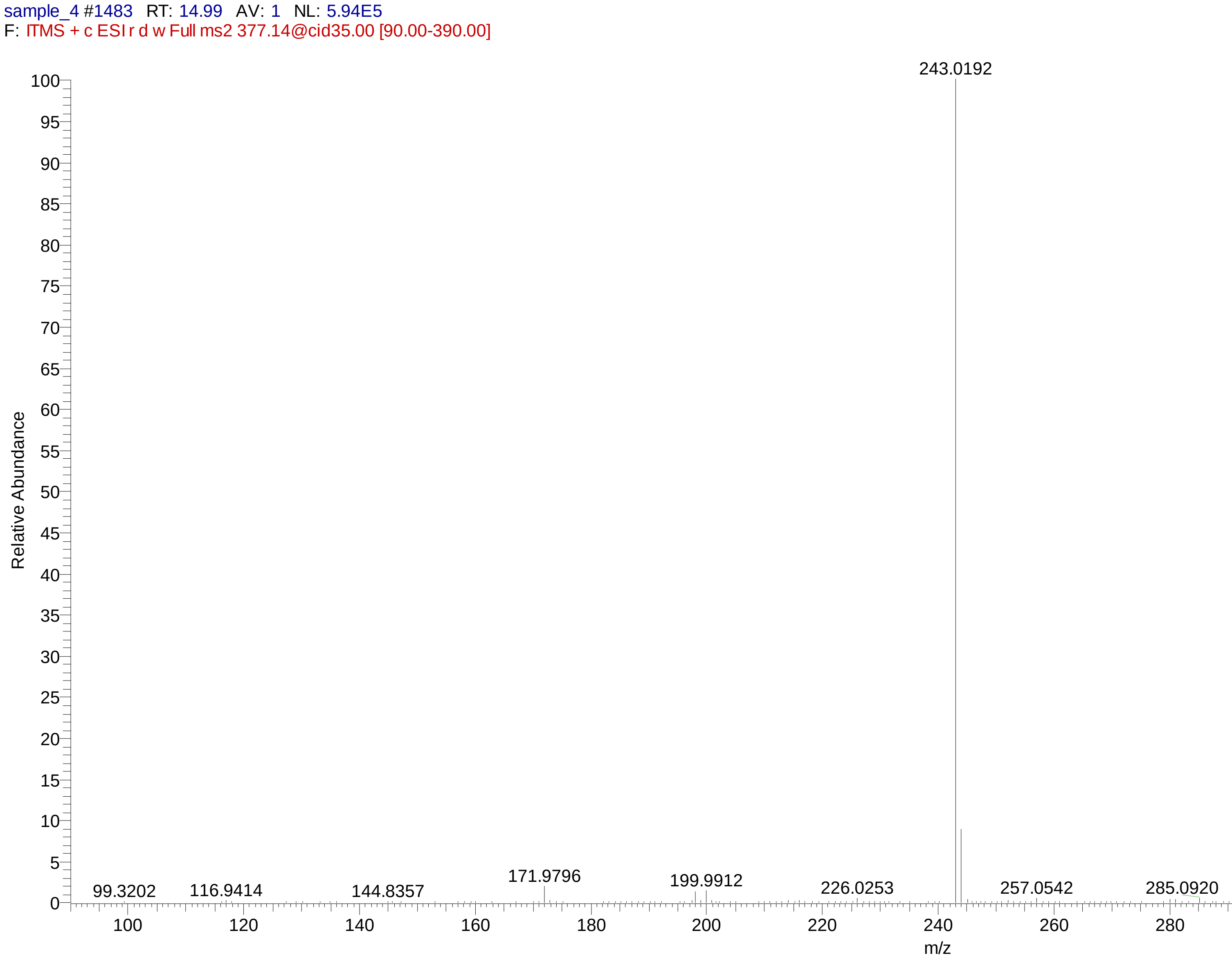

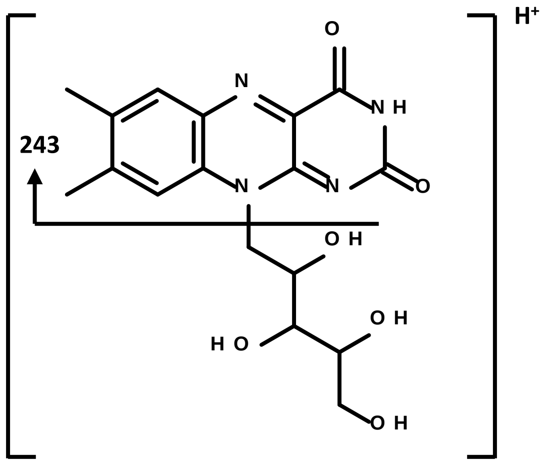

Compound 4	Formula	Ionspecies	*m/z*	RT [min]	Reference
Indoleacetic acid	$C_{10}H_9NO_2$	$[M+H]^+$	176.0706	21.00	https://www.mzcloud.org/DataViewer#Creference445#T799#c#105167

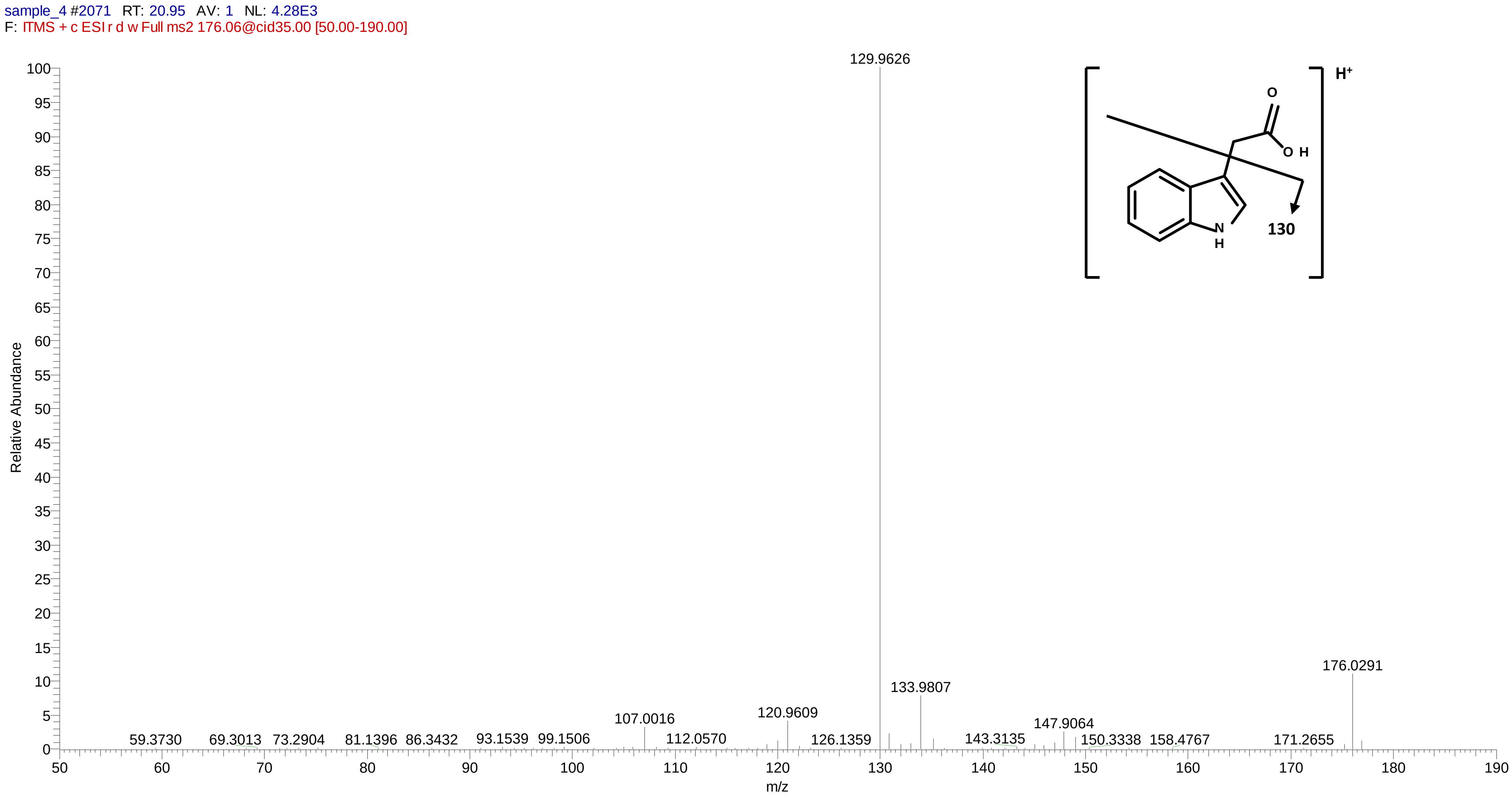

Compound 5	Formula	Ionspecies	*m/z*	RT [min]	Reference
Genistein/Galangin	$C_{15}H_{10}O_5$	$[M+H]^+$	271.0601	26.39	https://www.mzcloud.org/DataViewer#Creference24#T57#c#11083

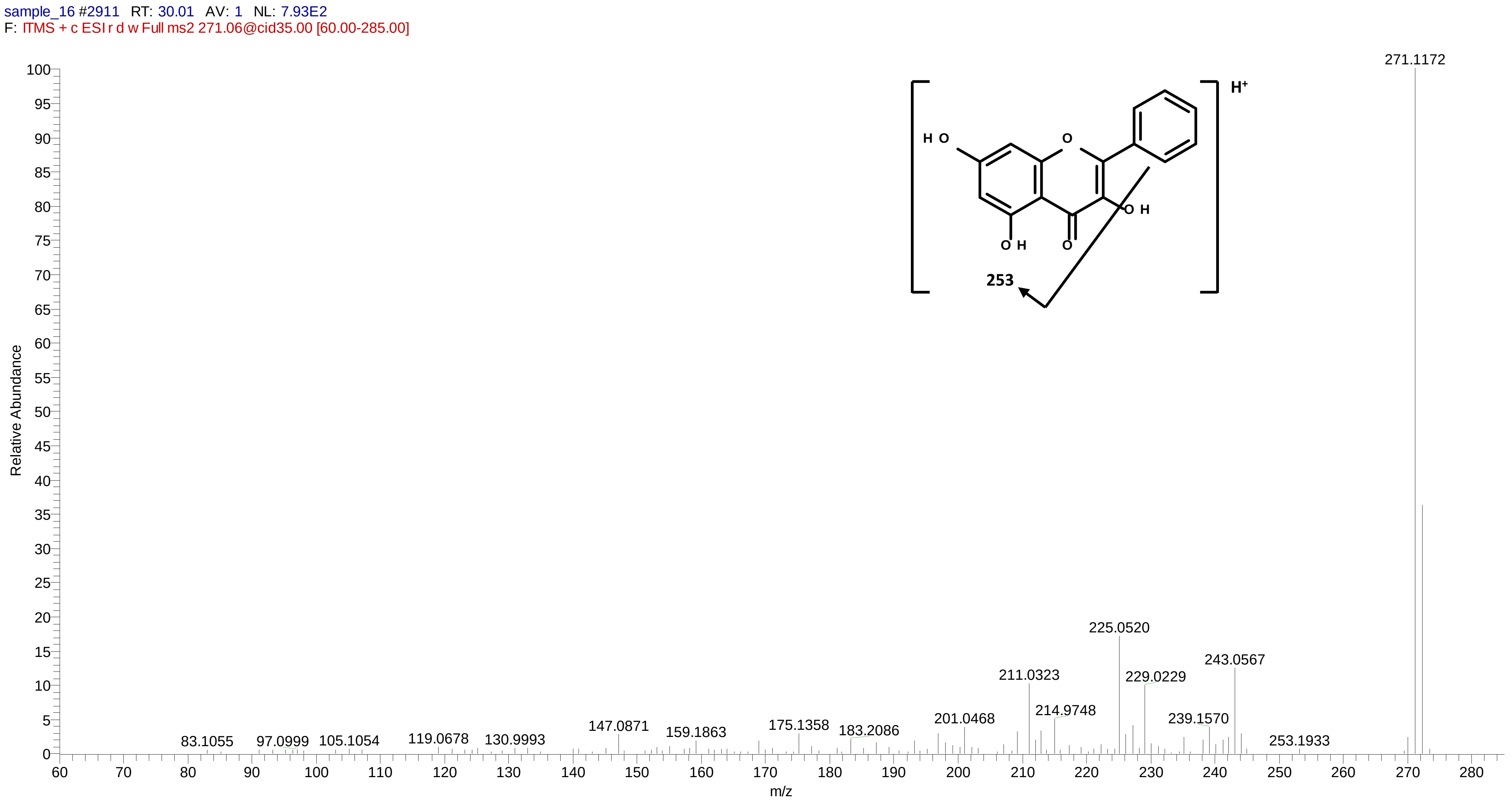

Compound 6	Formula	Ionspecies	*m/z*	RT [min]	Reference
Leucine/Isoleucine	$C_6H_{13}NO_2$	$[M+H]^+$	132.1019	4.26	https://www.mzcloud.org/DataViewer#Creference6#T17#c#401581

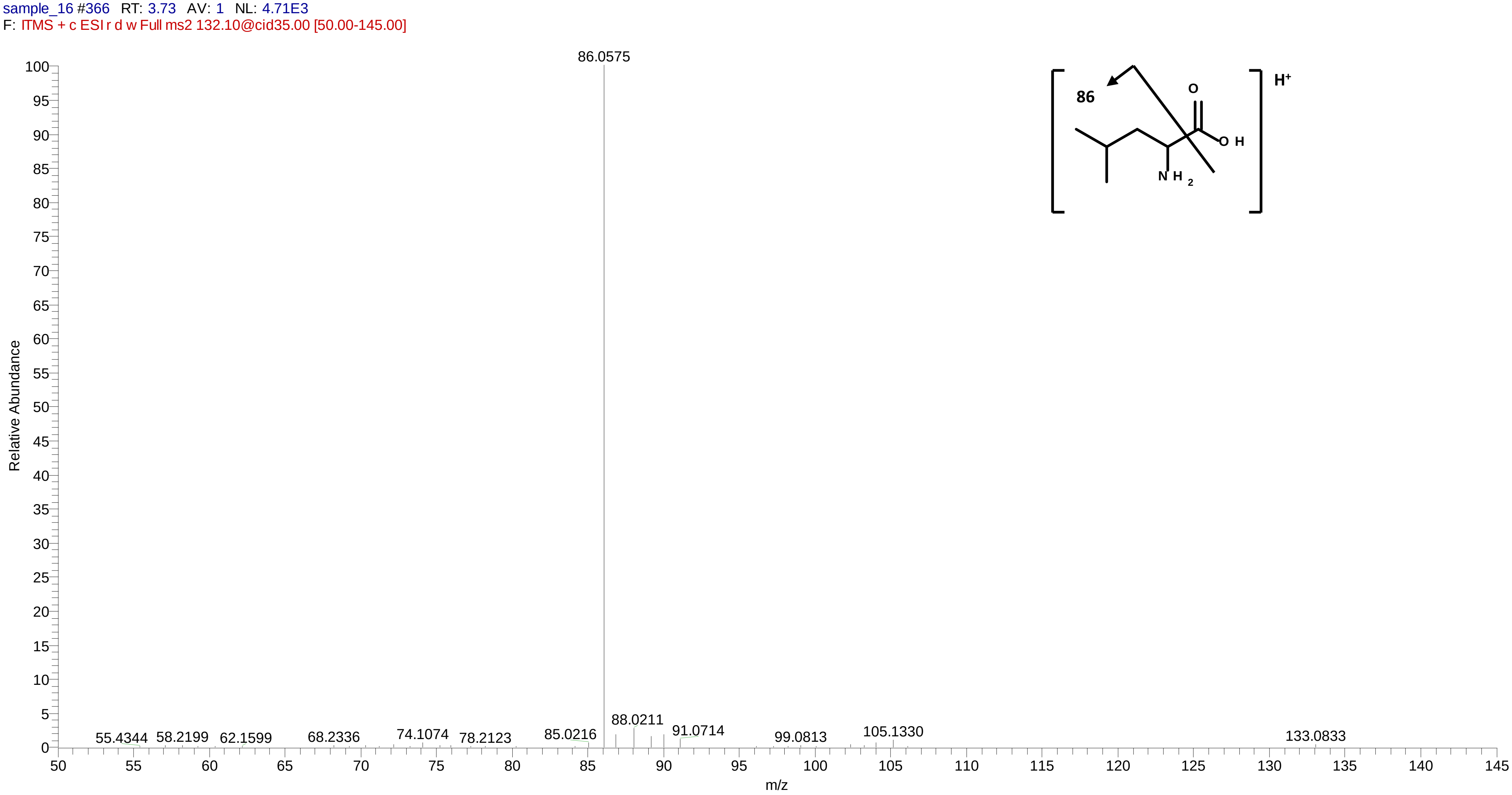

Compound 7	Formula	Ionspecies	*m/z*	RT [min]	Reference
Acetyl carnitine	$C_9H_{17}NO_4$	$[M+H]^+$	204.123	2.80	https://www.mzcloud.org/DataViewer#Creference879#T1526#c#186875

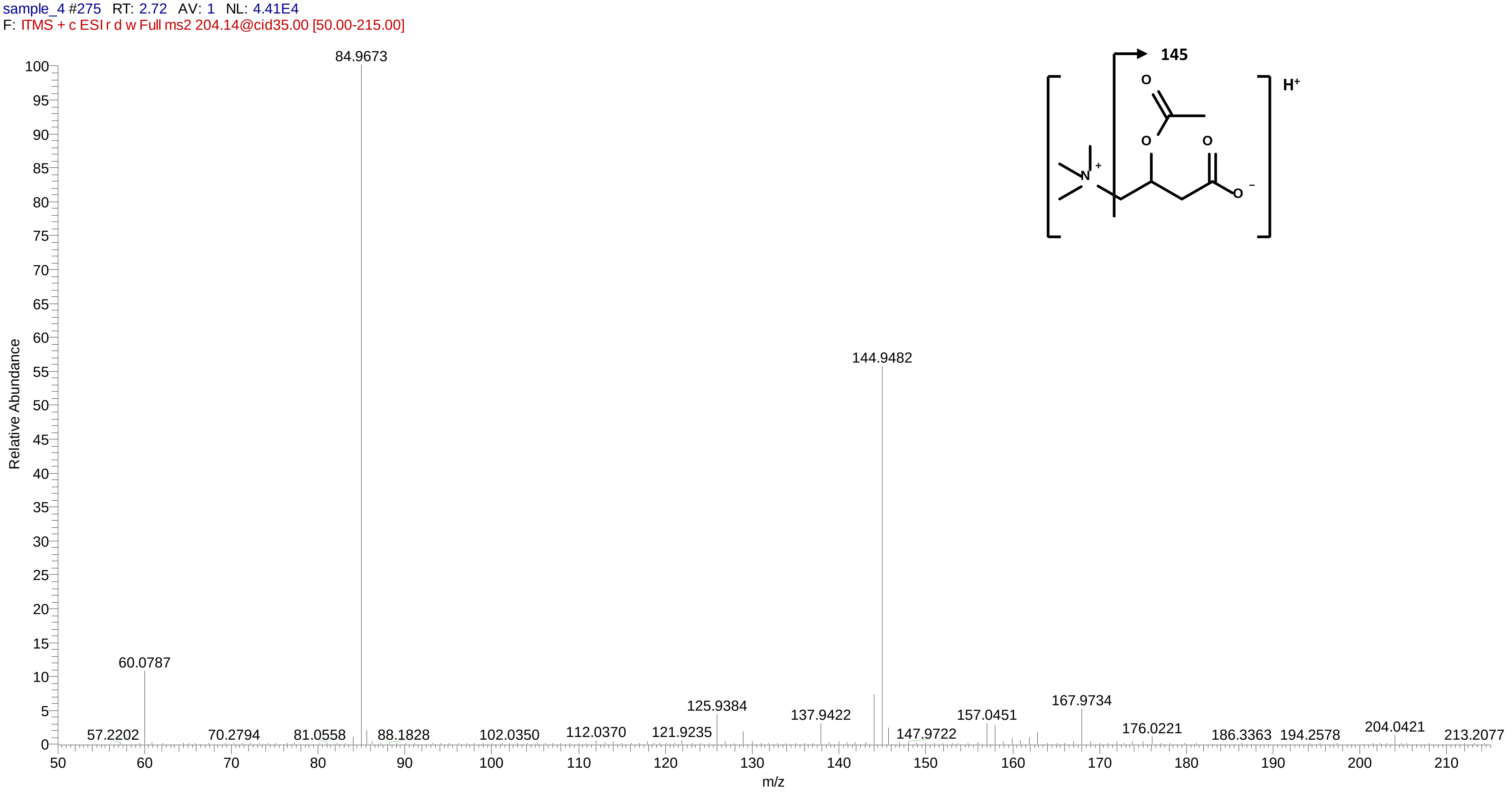

Compound 8	Formula	Ionspecies	*m/z*	RT [min]	Reference
Pantothenic acid	$C_9H_{17}NO_5$	$[M+H]^+$	220.118	7.02	https://www.mzcloud.org/DataViewer#Creference536#T981#c#130088

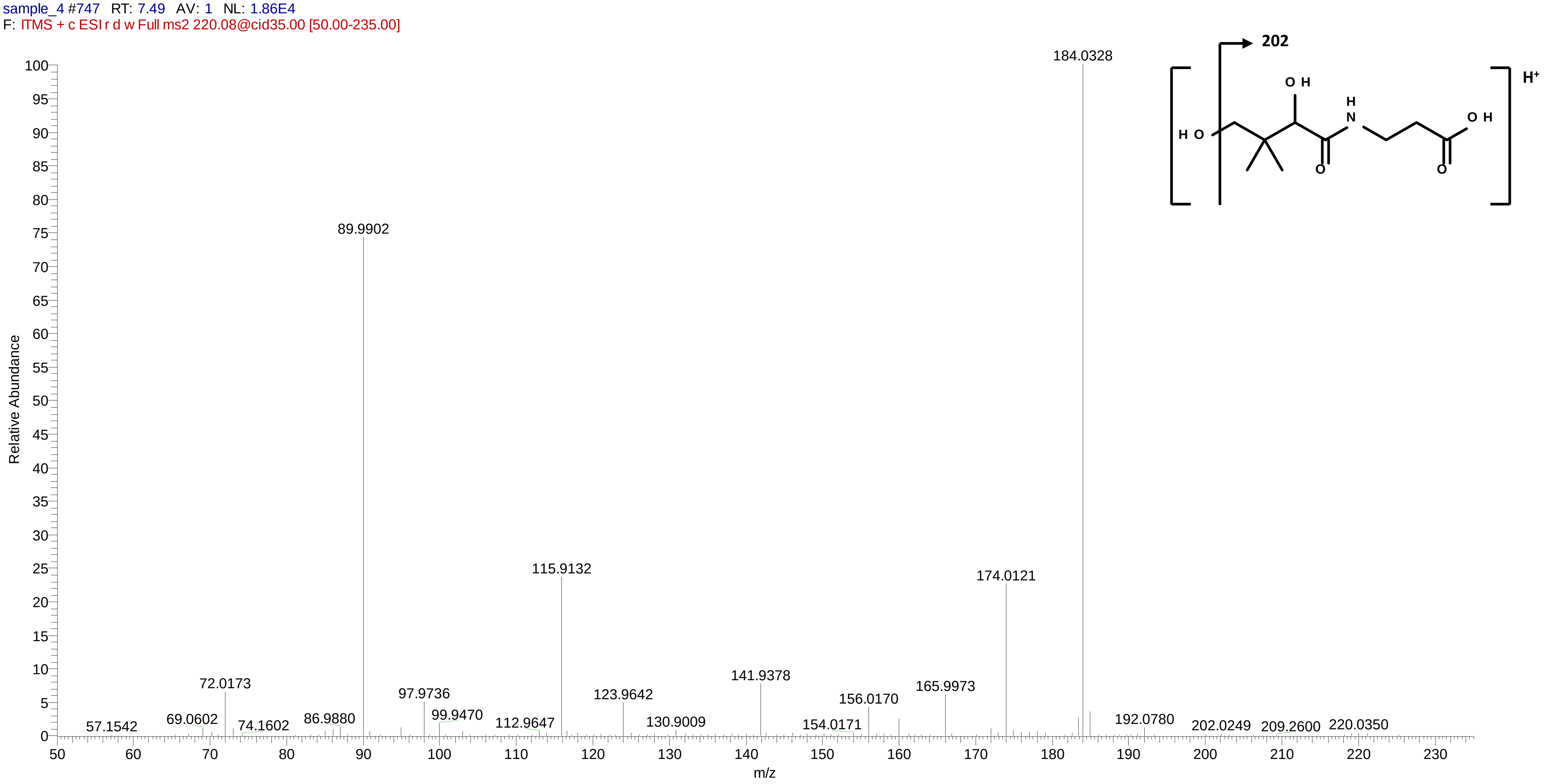

Compound 9	Formula	Ionspecies	*m/z*	RT [min]	Reference
Lysine	$C_6H_{14}N_2O_2$	$[M+H]^+$	147.1128	4.26	Was not fragmented

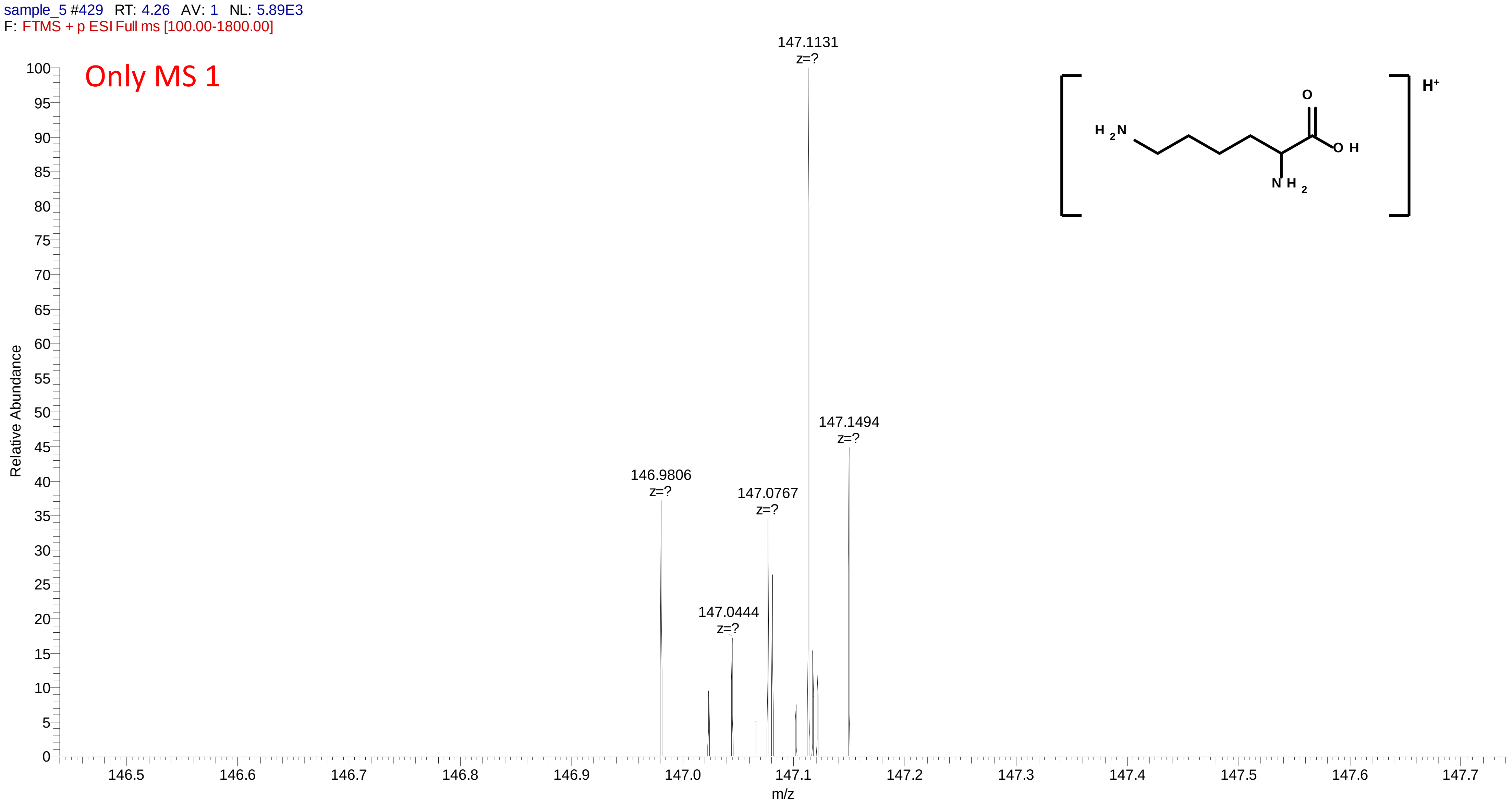

Compound 10	Formula	Ionspecies	*m/z*	RT [min]	Reference
Unknown1 putative phenolic	$C_{14}H_{12}O_4$	$[M+H]^+$	245.0808	26.06	No reference available

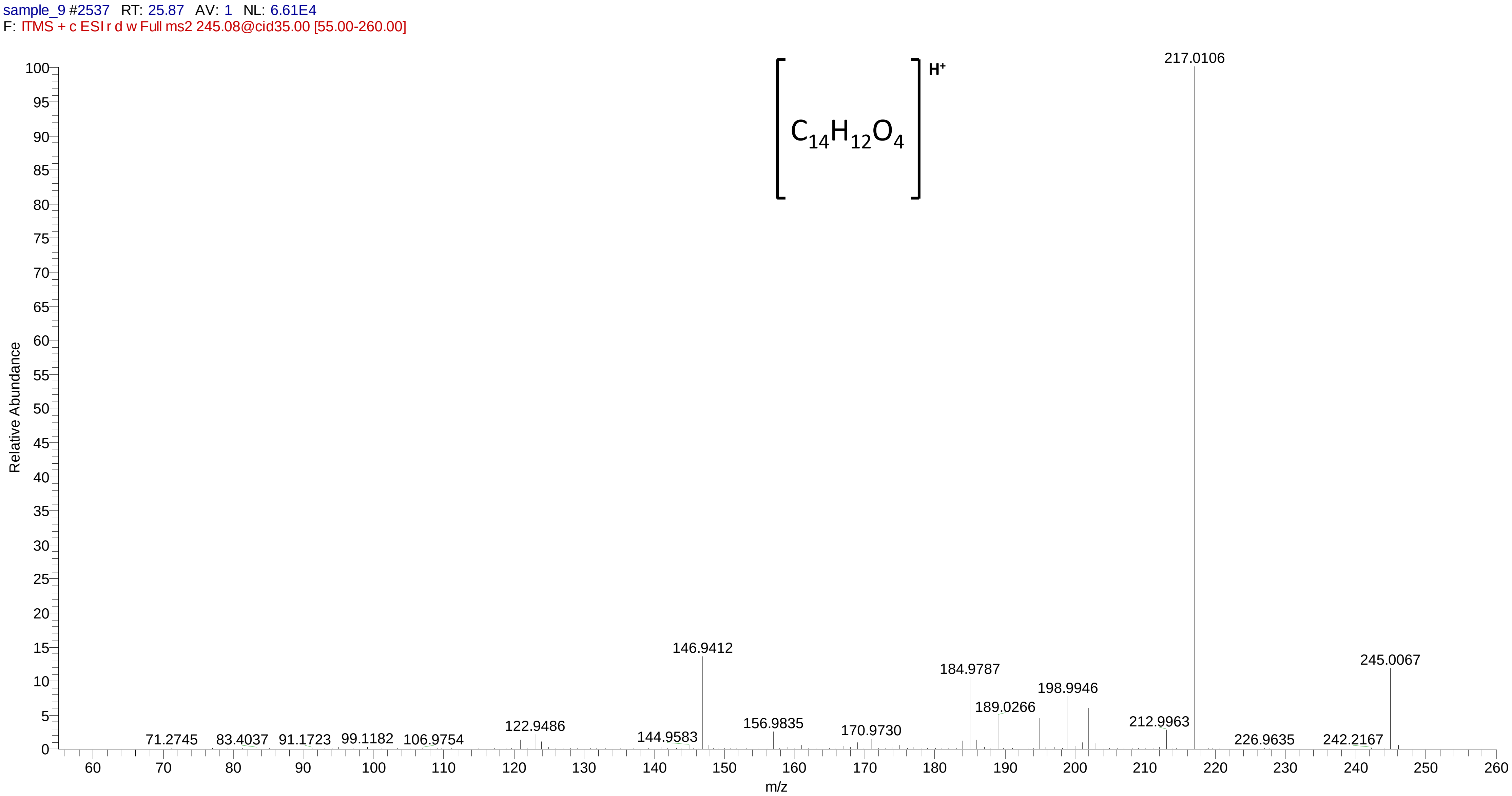

Compound 11	Formula	Ionspecies	*m/z*	RT [min]	Reference
Unknown2 putative phenolic hexosyl rhamnoside	$C_{26}H_{32}O_{13}$	$[M+H]^+$	553.1916	19.90	No reference available

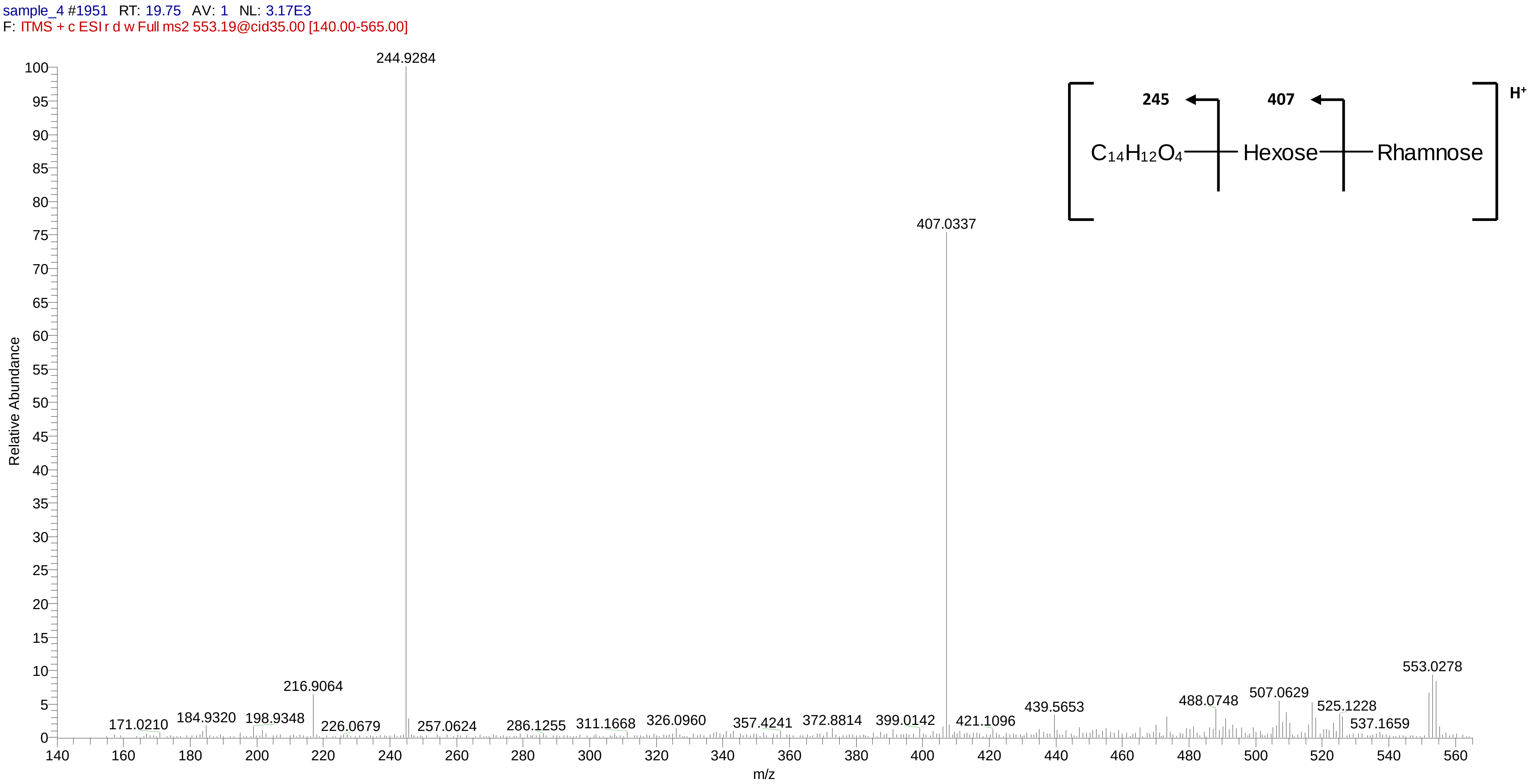

Compound 12	Formula	Ionspecies	*m/z*	RT [min]	Reference
Unknown3 putative phenolic hexosyl rhamnoside	$C_{26}H_{32}O_{13}$	$[M+H]^+$	553.1916	19.22	No reference available

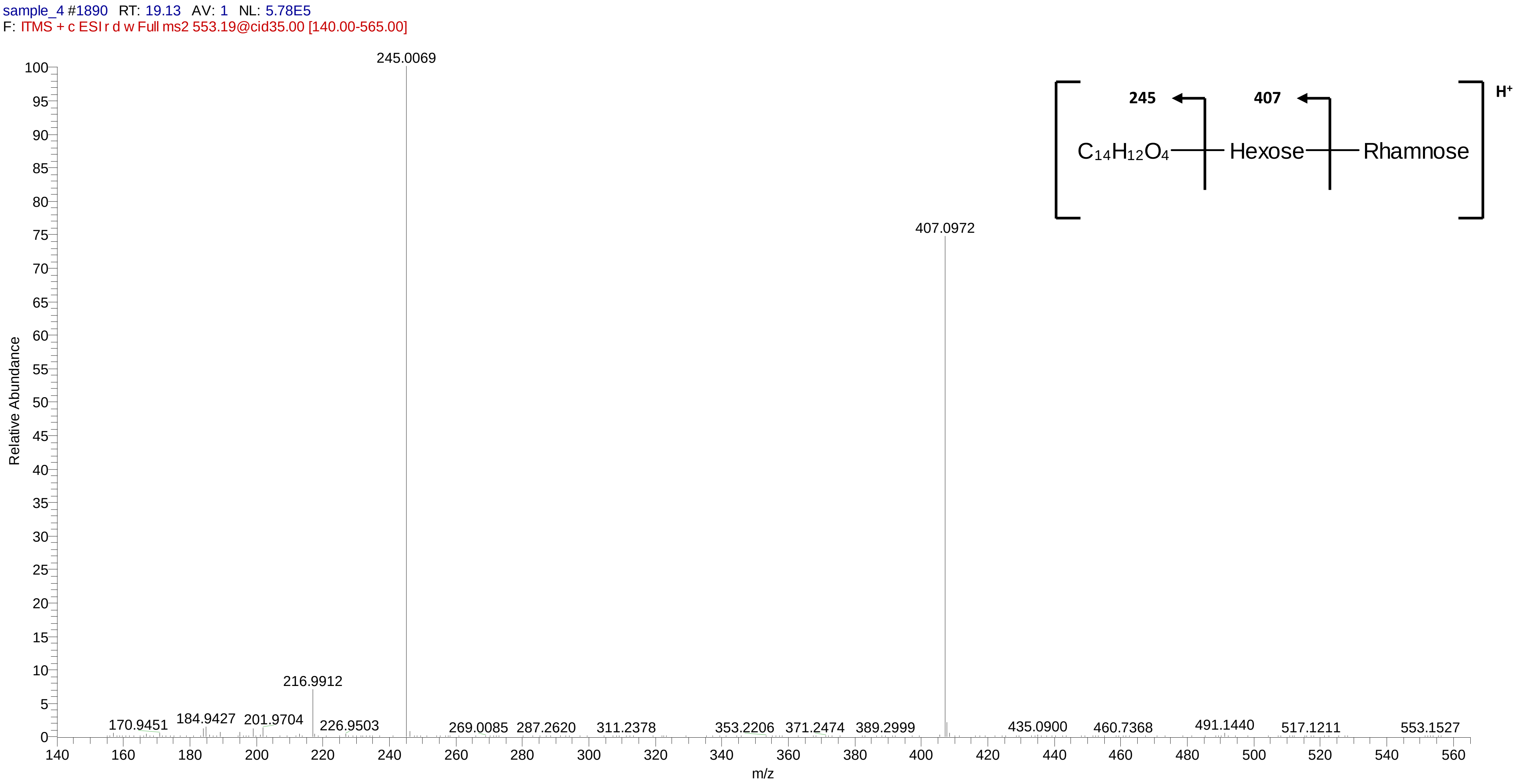

**Compound 13**	**Formula**	**Ionspecies**	***m/z***	**RT [min]**	**Reference**
Unknown4 putative phenolic hexosyl rhamnoside dimer	$C_{52}H_{64}O_{26}$	$[M+H]^+$	1105.3759	19.22	No reference available

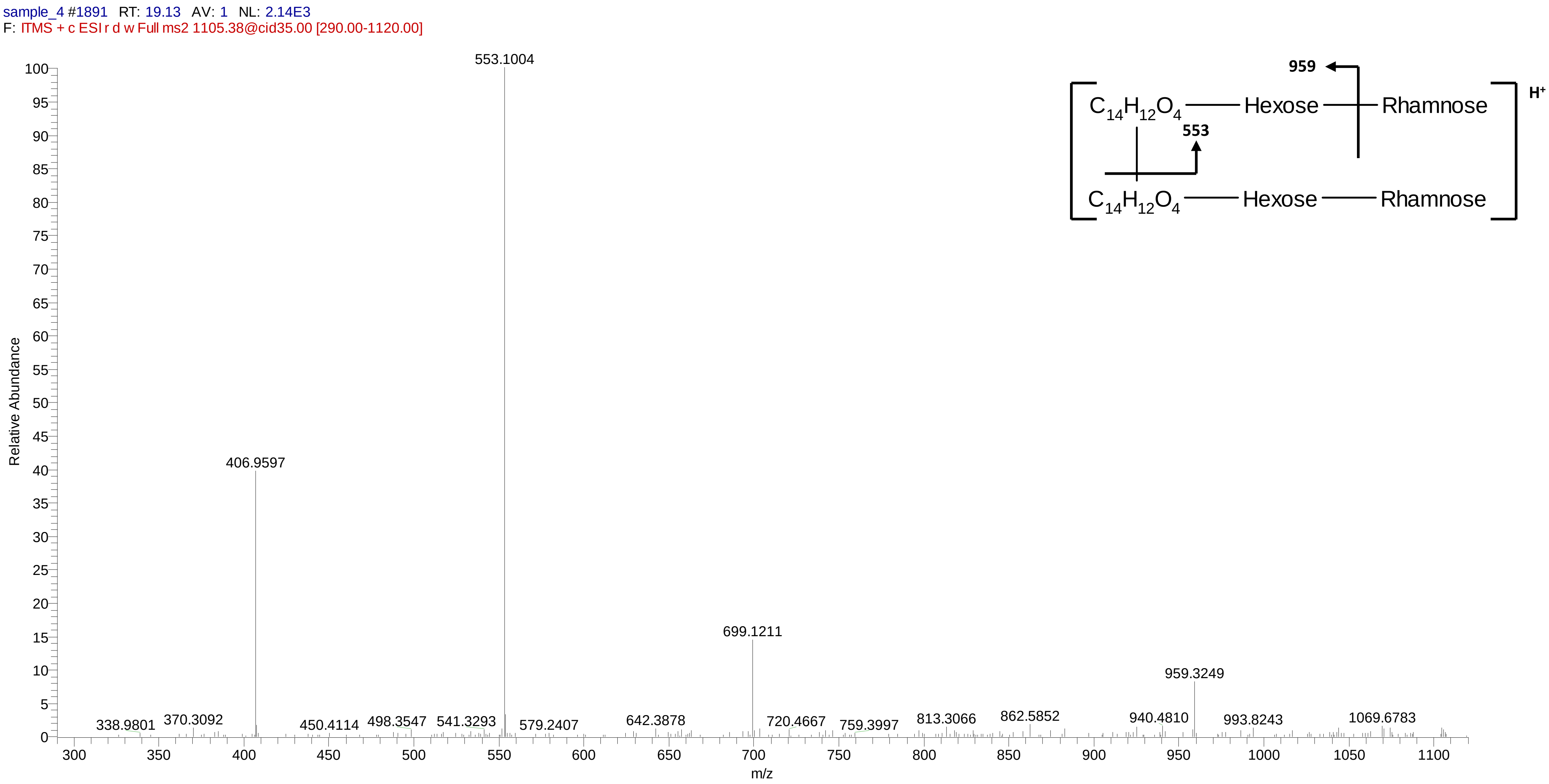

Compound 14	Formula	Ionspecies	*m/z*	RT [min]	Reference
Unknown5 putative phenolic hexoside	$C_{20}H_{22}O_9$	$[M+H]^+$	407.1337	19.31	No reference available

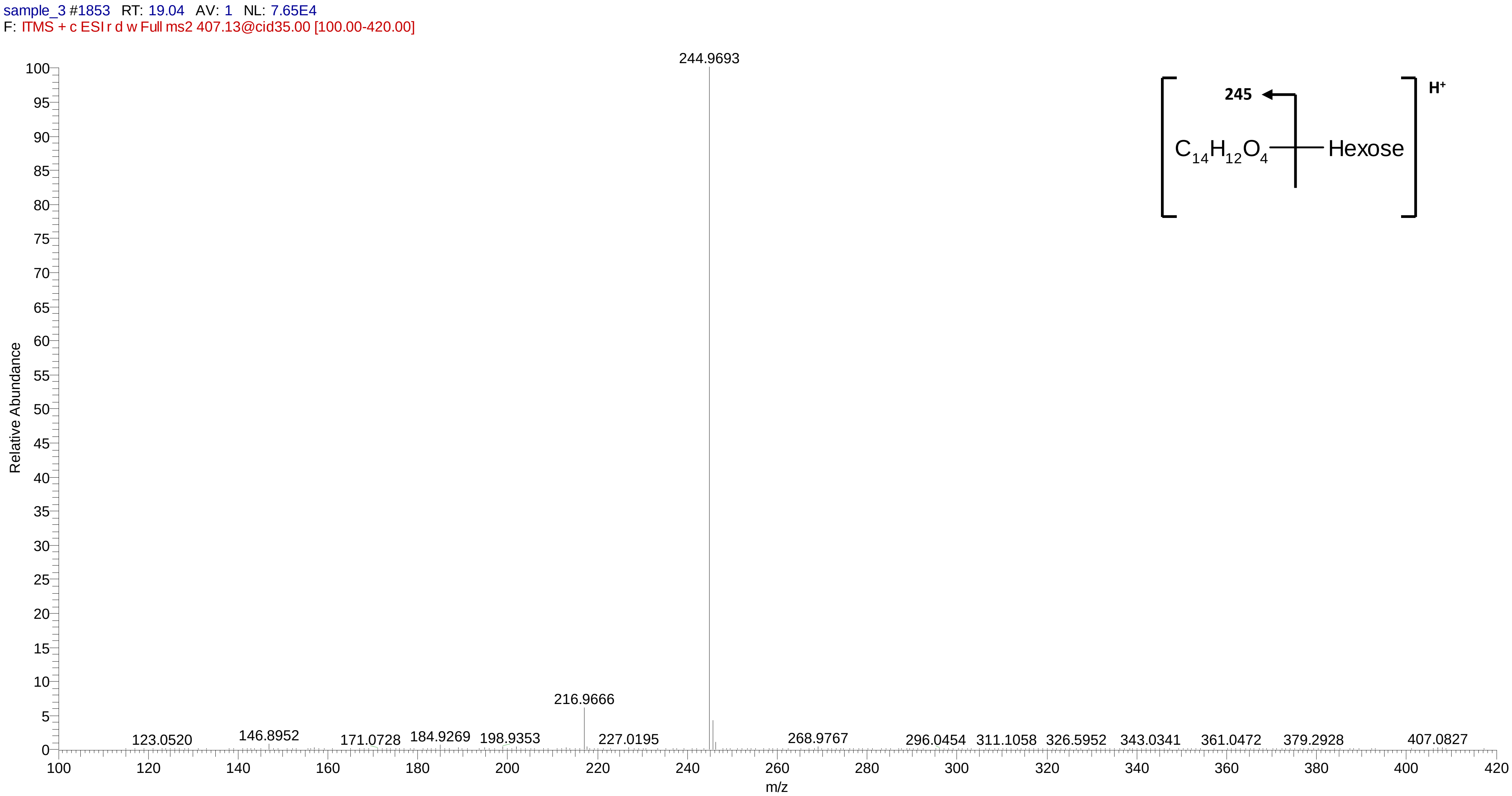

Compound 15	Formula	Ionspecies	*m/z*	RT [min]	Reference
Unknown6 putative phenolic hexoside dimer	$C_{40}H_{44}O_{18}$	$[M+H]^+$	813.26	19.45	No reference available

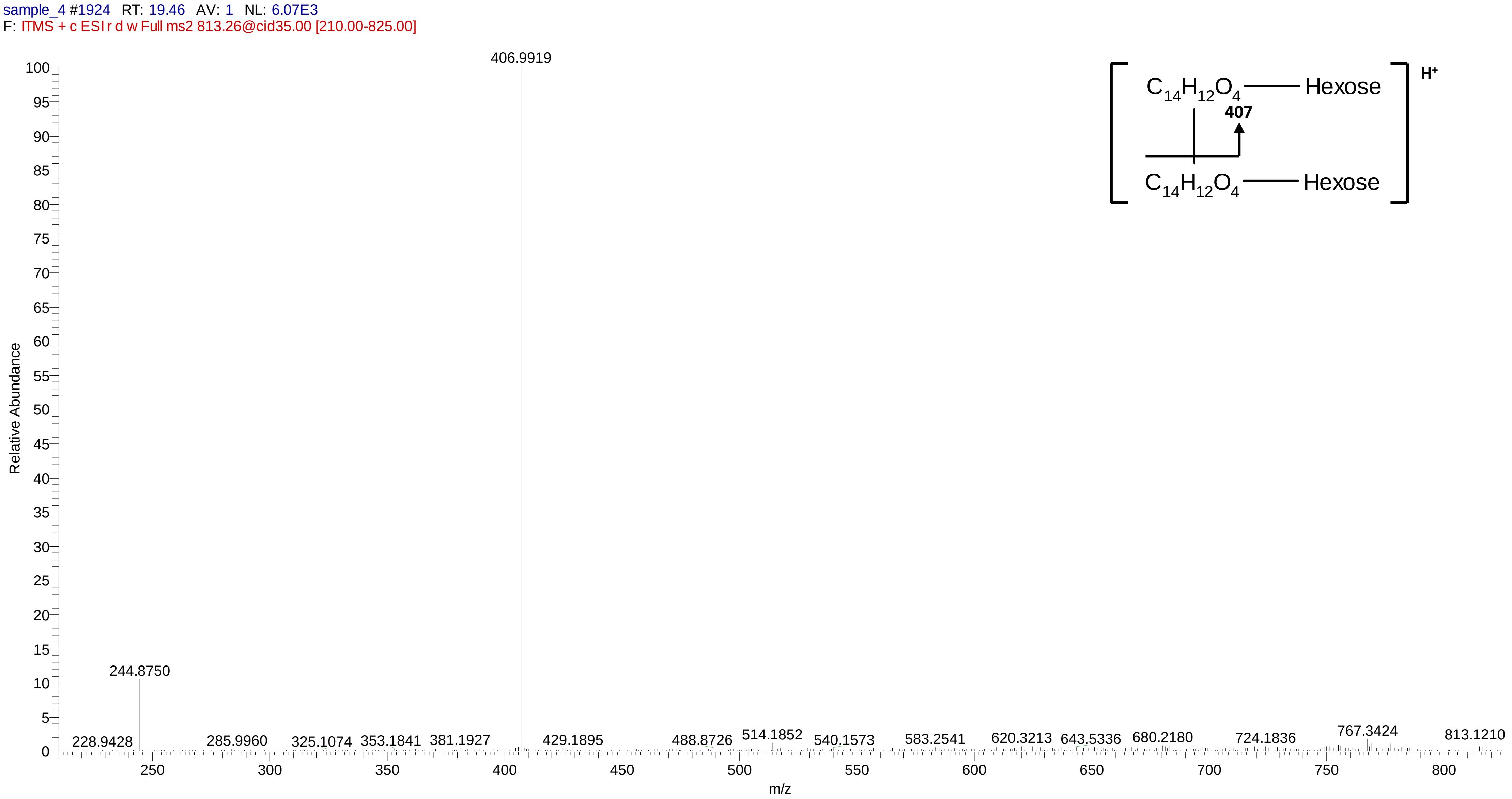

Compound 16	Formula	Ionspecies	*m/z*	RT [min]	Reference
Unknown7 putative phenolic dimer	$C_{28}H_{24}O_8$	$[M+H]^+$	489.1544	25.45	No reference available

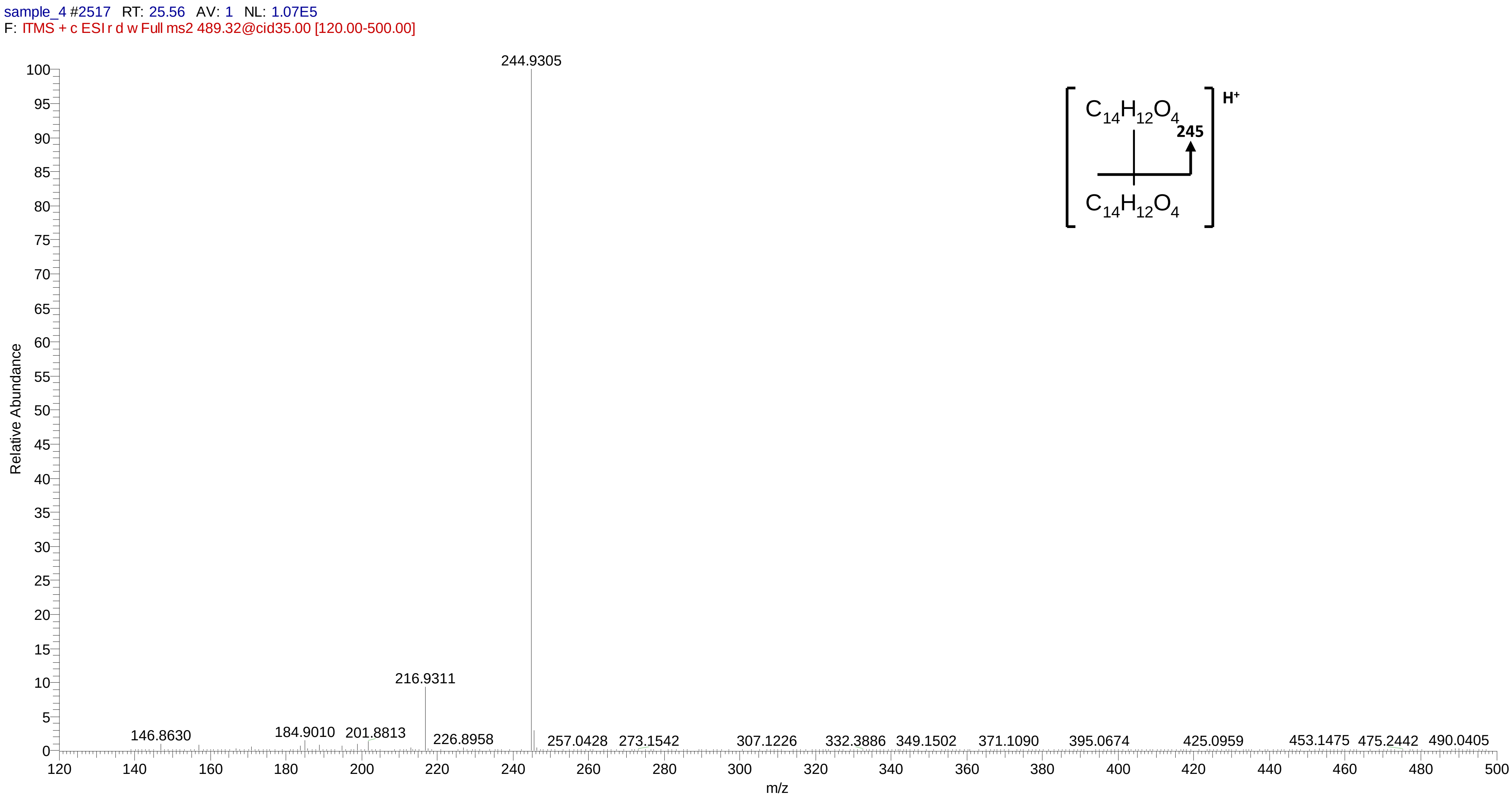

Compound 17	Formula	Ionspecies	*m/z*	RT [min]	Reference
Unknown8 putative phenolic dimer	$C_{28}H_{24}O_8$	$[M+H]^+$	489.1544	26.23	No reference available

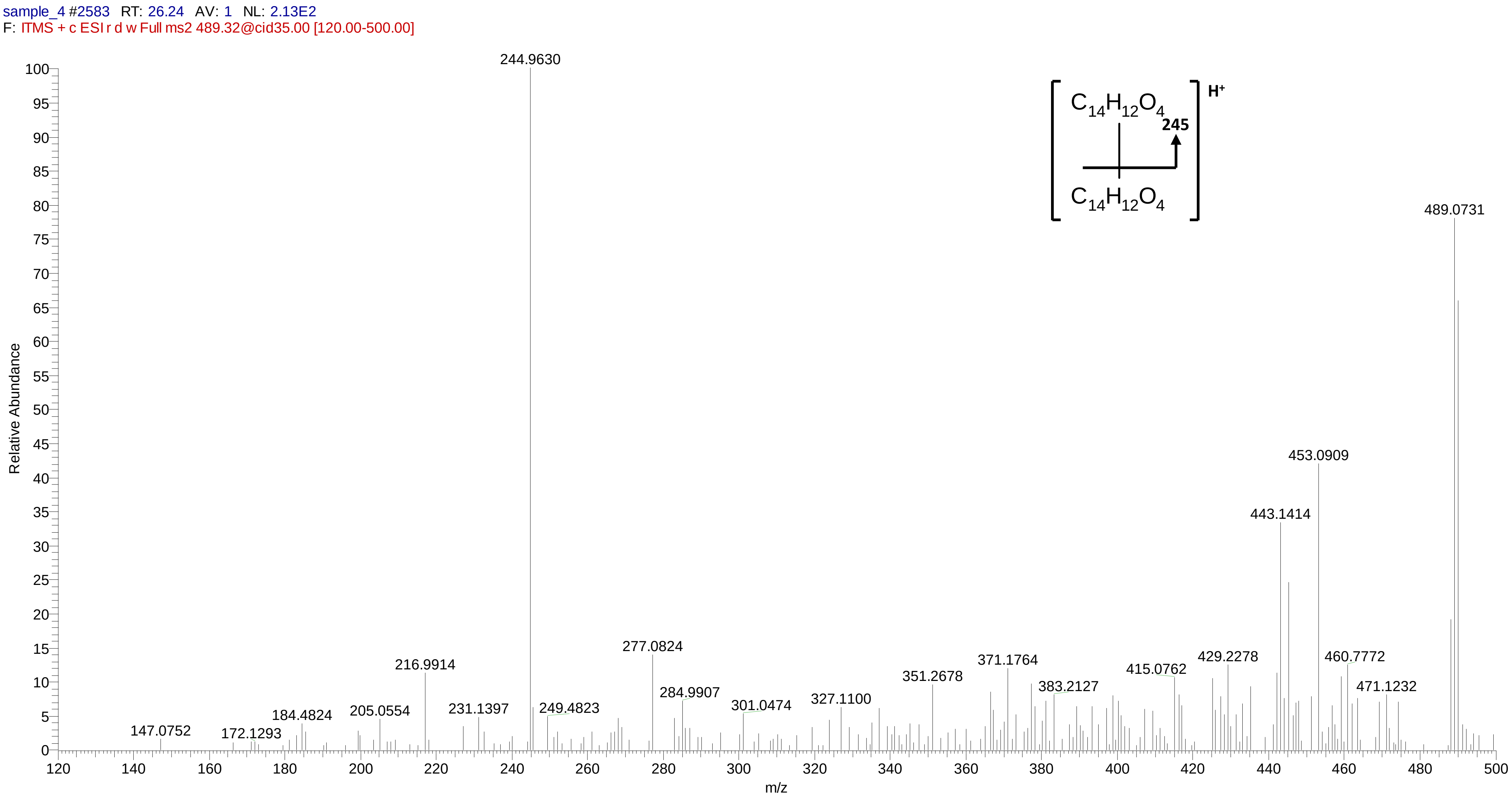

Supplement: Supplementary file 1 — Supplementary file1 Fig. S1: Pictures of the experimental setup in the glasshouse.Fig. S2: (a) Orthogonal partial least squares discriminant analysis (OPLS – DA) was performed considering all the compounds identified using UV-HPLC under control and stress condition in pearl millet genotypes (843-22B and ICTP8203). (b) Plot representing loadings of principal component analysis.Fig. S3: (a) Principal component analysis (PCA) of all the primary metabolites identified in the exudates of pearl millet genotypes (843-22B and ICTP 8203).Fig. S4: Principal component analysis (PCA) of the m/z features identified using in-house software mzFun (a) PCA of the m/z features identified from positive ion mode measurements (b) PCA of the m/z features identified from negative ion mode measurements.Fig. S5: Fragmentation spectra of the 17 secondary metabolites manually annotated. (PDF 1141 KB) [file 374_2021_1578_MOESM1_ESM.pdf]
